# Structural magnetic resonance imaging in dystonia: A systematic review of methodological approaches and findings

**DOI:** 10.1111/ene.15483

**Published:** 2022-07-22

**Authors:** Claire L. MacIver, Chantal M. W. Tax, Derek K. Jones, Kathryn J. Peall

**Affiliations:** ^1^ Neuroscience and Mental Health Research Institute Division of Psychological Medicine and Clinical Neurosciences Cardiff University School of Medicine Cardiff UK; ^2^ Cardiff University Brain Imaging Centre (CUBRIC) Cardiff University Cardiff UK; ^3^ Image Sciences Institute University Medical Center Utrecht Utrecht The Netherlands

**Keywords:** diffusion MRI, dystonia, movement disorders, MRI, systematic review

## Abstract

**Background and purpose:**

Structural magnetic resonance techniques have been widely applied in neurological disorders to better understand tissue changes, probing characteristics such as volume, iron deposition and diffusion. Dystonia is a hyperkinetic movement disorder, resulting in abnormal postures and pain. Its pathophysiology is poorly understood, with normal routine clinical imaging in idiopathic forms. More advanced tools provide an opportunity to identify smaller scale structural changes which may underpin pathophysiology. This review aims to provide an overview of methodological approaches undertaken in structural brain imaging of dystonia cohorts, and to identify commonly identified pathways, networks or regions that are implicated in pathogenesis.

**Methods:**

Structural magnetic resonance imaging studies of idiopathic and genetic forms of dystonia were systematically reviewed. Adhering to strict inclusion and exclusion criteria, PubMed and Embase databases were searched up to January 2022, with studies reviewed for methodological quality and key findings.

**Results:**

Seventy‐seven studies were included, involving 1945 participants. The majority of studies employed diffusion tensor imaging (DTI) (*n* = 45) or volumetric analyses (*n* = 37), with frequently implicated areas of abnormality in the brainstem, cerebellum, basal ganglia and sensorimotor cortex and their interconnecting white matter pathways. Genotypic and motor phenotypic variation emerged, for example fewer cerebello‐thalamic tractography streamlines in genetic forms than idiopathic and higher grey matter volumes in task‐specific than non‐task‐specific dystonias.

**Discussion:**

Work to date suggests microstructural brain changes in those diagnosed with dystonia, although the underlying nature of these changes remains undetermined. Employment of techniques such as multiple diffusion weightings or multi‐exponential relaxometry has the potential to enhance understanding of these differences.

## Graphical abstract

This article systematically reviews the literature applying structural magnetic resonance imaging in dystonia. An overview is provided of methodological approaches and common implicated regions. Key grey matter motor control regions and their interconnecting white matter most consistently demonstrate differences compared to control populations.

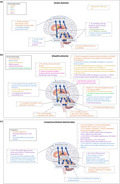

## INTRODUCTION

Dystonia is a movement disorder involving repetitive or sustained muscle contractions leading to abnormal posturing, with an estimated prevalence of 120/100,000 population [1]. Clinical presentation is heterogeneous, involving single or multiple muscle groups (focal, segmental or generalized), with genetic or idiopathic aetiology, and of childhood or adult onset [2]. Animal models have implicated the cerebellum and basal ganglia in pathogenesis, demonstrating cerebellar Purkinje cell abnormalities, including ectopic dendritic spines and aberrant firing patterns, as well as disrupted striatal gamma aminobutyric acid (GABA) and dopamine neurotransmission [3]. Human postmortem studies support this, with patchy cerebellar cell loss and torpedo bodies in cervical dystonia [4].

**FIGURE 1:**
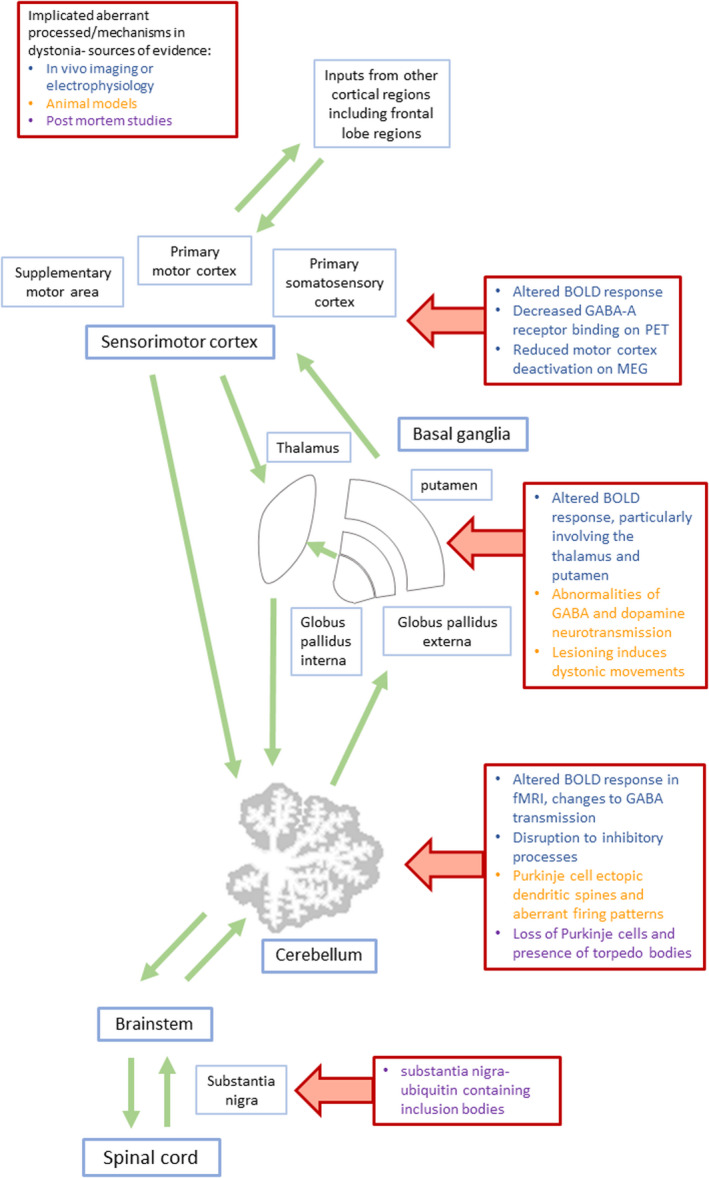
Motor control pathways and proposed pathological mechanisms in dystonia. Differences are relative to healthy controls unless otherwise stated [Colour figure can be viewed at wileyonlinelibrary.com]

In vivo studies show network‐based disruption to cerebral motor control pathways in dystonia [5], with differences observed in the primary sensorimotor cortex, putamen, thalamus and cerebellum in functional magnetic resonance imaging (fMRI) and electrophysiological studies, the latter also implicating disruption to normal inhibitory processes [6]. Inhibitory/excitatory imbalances are also suggested by MR spectroscopy and radionucleotide imaging, with changes to GABA neurotransmission observed in cerebellar and sensorimotor cortices [5, 7] (Figure 1). Despite this, standard clinical structural MR sequences have not demonstrated gross abnormalities, suggesting changes may be at the microstructural level.

Multiple MRI approaches are used to derive information regarding brain structure in medical research. Diffusion MRI (dMRI) can probe tissue microstructure based on the degree of freedom of molecular movement, deriving properties including mean diffusivity (MD, the overall freedom of diffusion), fractional anisotropy (FA, the degree of orientational preference), axial diffusivity (AxD, the apparent diffusion coefficient along the dominant diffusion axis) and radial diffusivity (RadD, the degree of diffusion in the plane perpendicular to the primary diffusion direction) (Figure 2.1a). Other approaches aim to identify subtle localized volume or size differences, with atlas‐based automatic or manual approaches to delineating brain regions and comparing between groups (Figure 2.1b). Thirdly, relaxometry methods aim to provide a quantitative measure of molecular relaxation following excitation with a radiofrequency pulse, inferring information relating to local tissue properties which can influence the speed of this relaxation. Common relaxometry approaches include T2* relaxometry, heavily influenced by susceptibility effects which can be particularly substantially induced by iron, and T2 relaxometry which more closely relates to water content (Figure 2.1c). Finally, magnetization transfer imaging investigates the effects of macromolecules on unbound free water, the signal for which decays too rapidly to measure directly, allowing signal sensitivity to tissue structures such as membranes and myelin (Figure 2.1d).

**FIGURE 2:**
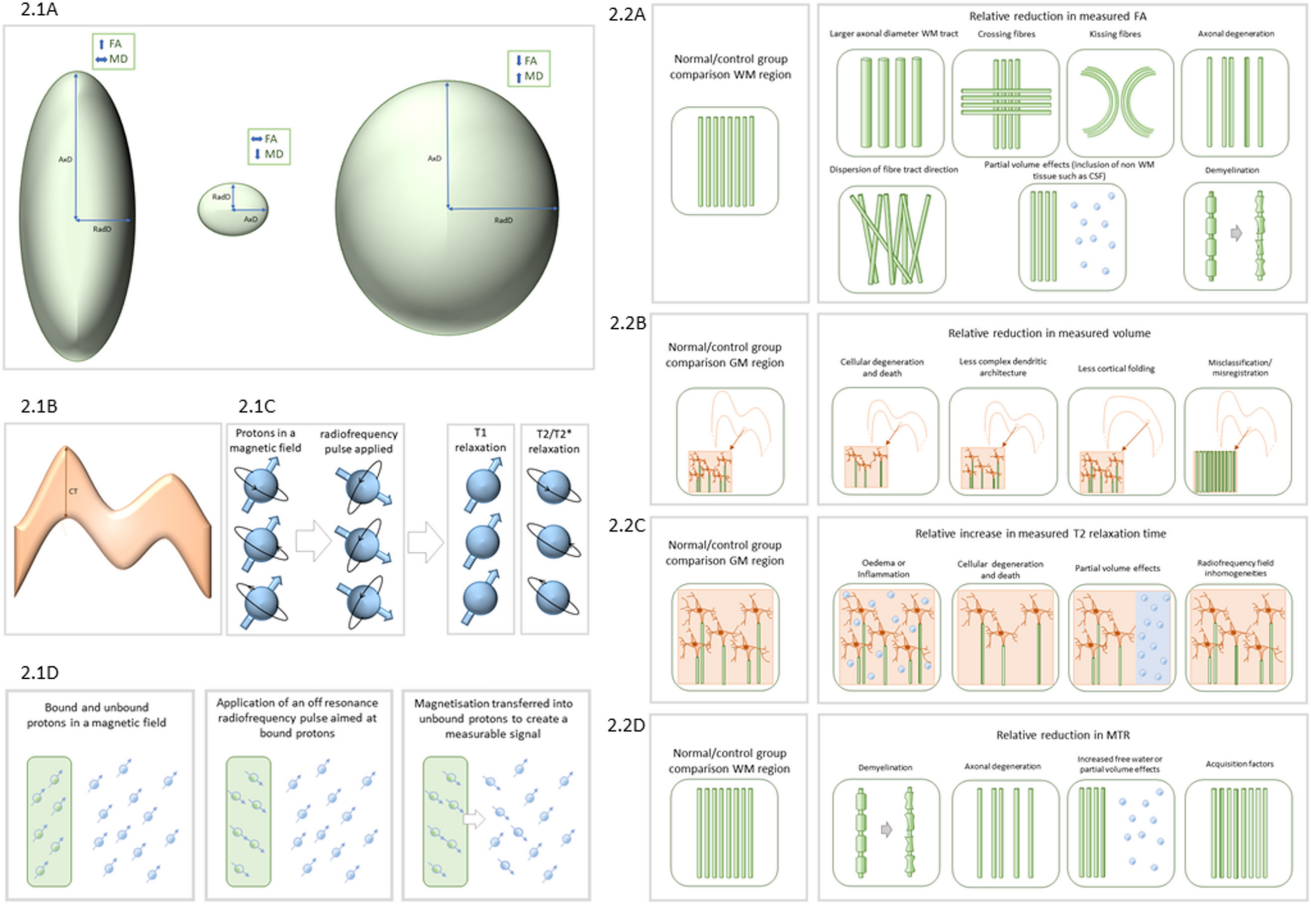
Common structural measures. 2.1 Examples of structural measurements. (1a) DTI properties with examples showing a relatively high FA and AxD, a lower FA and MD, and a low FA but a high MD. (1b) Examples of volume/size‐based measures with the shaded area representing the measured volume for the segmented region and the arrow indicating the cortical thickness (CT). (1c) Relaxometry examples showing the hydrogen ions aligned in the magnetic field of an MR scanner, with the application of a radiofrequency pulse causing them to come out of alignment with the magnetic field (blue arrows) and their spins coming out of alignment with each other (black arrows). T1 is the time taken for longitudinal relaxation (i.e., the blue arrow to return to alignment with the main magnetic field) and T2 is the time taken for transverse relaxation (i.e., the black arrows to return to being out of phase with each other), with T2* being additionally influenced by local magnetic field differences. Proton density is a measure of how densely packed the protons are. (1d) Magnetization transfer imaging, showing bound and unbound protons in a magnetic field, with a radiofrequency pulse aimed mainly at bound protons applied, and then the transfer of this magnetization to the unbound protons which produces a measurable signal; this is MT weighting. MTR is the difference between an acquisition with and without this off‐resonance pulse. 2.2 Examples of factors influencing structural measures for FA (2a), volumetry (2b), T2 relaxometry (2c) and magnetization transfer ratio (2d) [Colour figure can be viewed at wileyonlinelibrary.com]

This review evaluates structural MRI studies used in the investigation of genetic and idiopathic forms of dystonia to date, with particular emphasis on methodological considerations including study design, imaging acquisition, pre‐processing and analysis methods. Our aim is to synthesize the breadth of work undertaken to date, to critically appraise the imaging methodological approaches used and to highlight consistent anatomical findings which may provide pathophysiological insights of dystonia.

## METHODS

In line with PRISMA guidelines, studies using structural brain MRI in genetic and idiopathic forms of dystonia were systematically reviewed. Embase and PubMed databases were searched for articles up to January 2022. The full search strategy is detailed in Appendix S1. Inclusion criteria were case‐controlled studies using structural MRI in the investigation of dystonia. Exclusion criteria included single case reports, no control cohort, secondary or psychogenic/functional dystonia, no use of structural MRI, deep brain stimulation studies using imaging only as a surgical planning tool, studies with minimal methodological detail, methodological testing, conference proceedings, review articles, where no full paper was available, not written in the English language. Abstracts were screened for inclusion and exclusion criteria by two investigators (C.M. and K.J.P.) working independently with further screening of the full text of the identified articles. Data were extracted from each of the studies to include type of dystonia, number of patients, patient demographics and phenotyping, imaging modality, imaging acquisition features, imaging pre‐processing steps, imaging analysis methodology and study findings. Overall risk of bias was assessed by two investigators (C.M. and K.J.P.) based on the risk of bias in non‐randomized studies (RoBANS) bias assessment tool [8] with additional consideration of specific imaging methodology features.

## RESULTS

### Structural MRI modalities

Seventy‐seven studies were identified, four of which were animal models of dystonia. Of the 73 human studies, 45 used dMRI, 39 size/volume‐based methodology, three relaxometry‐based approaches and one magnetization transfer imaging; 14 studies combined multiple approaches. In interpreting the findings of these studies multiple factors have the potential to impact image and data quality, and ultimately the study findings, including the image acquisition approach, data pre‐processing and analysis methodology (Appendices S2 and S3).

#### Imaging acquisition considerations

##### Field strength

A stronger magnetic field (measured in tesla, T) provides higher signal to noise ratio, enabling higher image resolution; however, stronger fields are limited by faster signal loss, exacerbation of inhomogeneities within the field (which can distort the measured signal) and potential tissue heating due to differing radiofrequency requirements. The studies identified used either 1.5 T (*n* = 16) [9, 10, 11, 12, 13, 14, 15, 16, 17, 18, 19, 20, 21, 22, 23, 24] or 3 T (*n* = 57) strength.

##### Voxel isotropy and size

Higher levels of voxel anisotropy (dimensions of differing sizes) and larger voxel sizes are potential confounding factors, with greater propensity to partial volume effects with inclusion of different tissue types within a single voxel (Figure 3.1a–1c). Smaller, isotropic voxels help to reduce (but not eliminate) these confounds. Eleven of the volumetric studies had either non‐isotropic voxels [16, 17, 22, 25, 26, 27, 28] or incomplete dimension data [14, 29, 30, 31]. Fifteen diffusion studies [32, 33, 34, 35, 36, 37, 38, 39, 40, 41, 42, 43, 44, 45, 46] and two relaxometry studies [47, 48] used isotropic voxels, five used dimensions that came close to isotropy (slice thickness <1.2× the in‐plane resolution) [11, 31, 49, 50, 51], 24 studies were predominantly limited by larger slice thickness increasing voxel size and anisotropy [9, 10, 12, 13, 14, 30, 52, 53, 54, 55, 56, 57, 58, 59, 60, 61, 62, 63, 64, 65, 66, 67, 68, 69] and three provided no voxel dimension detail [15, 29, 70].

**FIGURE 3:**
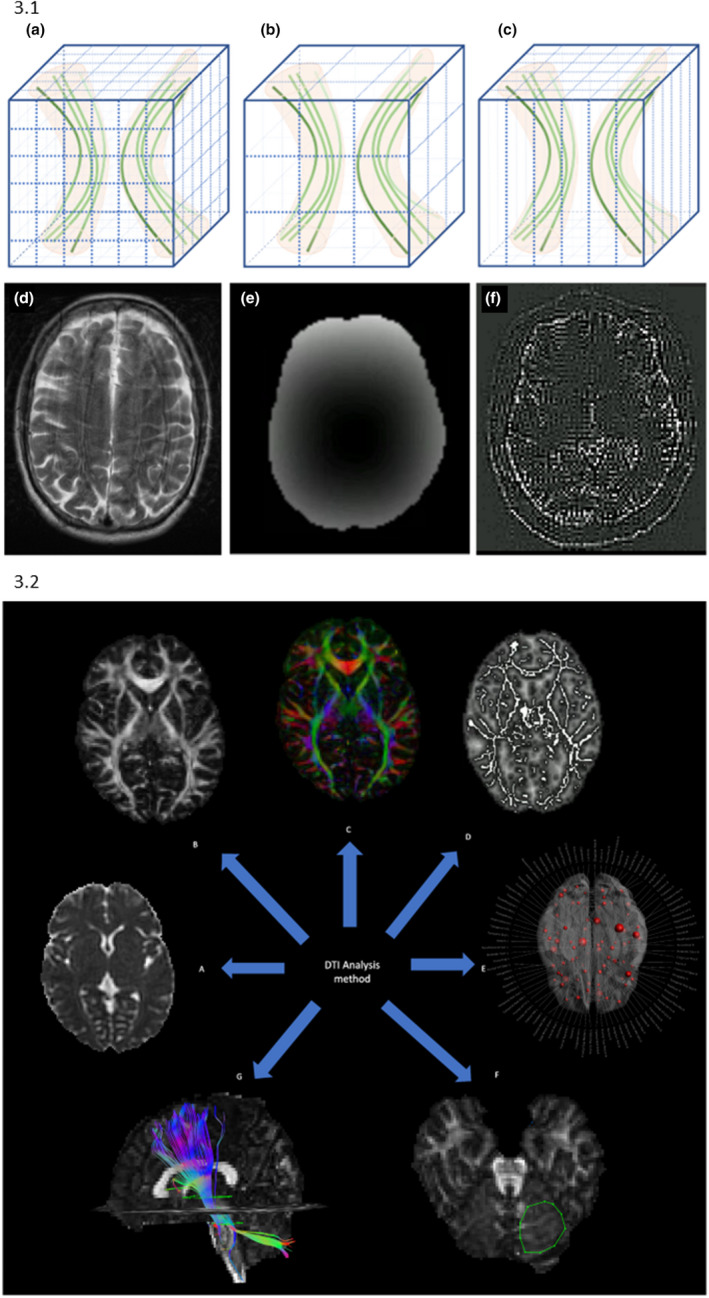
Structural imaging approaches. 3.1 Examples of factors influencing measured parameters. (1a–1c) Examples of the effect of differing voxel size and anisotropy on partial volume effect in white matter, with (1a) showing small isotropic voxels, (1b) larger voxels and (1c) anisotropic voxels, with more inclusion of tracts with different orientations in the larger and anisotropic voxels, which would influence the fractional anisotropy (FA) measured. (1d) Example of the effect of motion on a T2‐weighted image, (1e) a gradient deviation map showing variation in the magnetic field and (1f) an example of the signal removed from the Gibbs ringing artifact in diffusion MRI. 3.2 DTI as an example of the range of potential analysis approaches: (2a) a map of MD (mean diffusivity) values, (2b) a map of FA values, (2c) T directional orientation colour‐coded FA map, (2d) tract‐based spatial statistics, (2e) graph theoretical analysis, (2f) a region of interest delineated in the cerebellum and (2g) an example of a tractography reconstruction using regions of interest to define way‐points along the tract [Colour figure can be viewed at wileyonlinelibrary.com]

##### Methodology‐specific acquisition features

There are additional methodologically specific important acquisition features. For dMRI studies, using more diffusion directions can improve the precision of the calculated diffusion tensor, and a greater degree of diffusion weighting (a higher *b* value) can enable assessment of more subtle diffusion properties but with the trade‐off of increased potential for artifacts and lower signal to noise ratio. Of the studies identified, the number of diffusion directions ranged from 6 (*n* = 5) [9, 10, 11, 12, 13] to ≥60 (*n* = 18) [14, 31, 33, 35, 37, 39, 42, 43, 44, 45, 49, 50, 51, 54, 57, 58, 59, 63], the remaining studies using an intermediate number of directions (*n* = 10) [30, 32, 34, 36, 38, 40, 41, 46, 52, 53, 55, 56, 60, 61, 62, 64, 65, 66, 67, 68, 70] or no details were provided (*n* = 1) [15]. The *b* values employed were in typical ranges of 700–1000 s/mm^2^, with five higher at 1100–1500 s/mm^2^ [38, 39, 49, 50, 51].

Amongst the volumetric studies, spatial resolution is important, with smaller voxel sizes reducing partial volume effects and enhancing delineation of anatomical borders, resulting in more accurate volumetric measures. Specifically, amongst the volumetric studies, the majority had dimensions of ≤1 mm^3^ (*n* = 27), with the rest having a maximum dimension of 1–1.5 mm (*n* = 7) [17, 22, 24, 25, 26, 27, 28], 1.5–2 mm (*n* = 1) [16] or not stated (*n* = 4) [14, 29, 30, 31].

With relaxometry, the number of echo times (TEs) or flip angles used to characterize the relaxation profile can influence estimation, with more data points and optimized spacing generally improving accuracy. This is particularly important for T2* acquisitions, as the gradient echo acquisition method is more greatly influenced by inhomogeneities in the magnetic field, resulting in a higher risk of noise influencing the measurements. For the T2* acquisitions the studies used six [69], eight [47] and 12 [48] different TEs; one study also assessed T2 relaxation time with four different TEs, and T1 and proton density (density of protons) with two flip angles [47]. The single magnetization transfer study [15] was based on the magnetization transfer ratio (MTR), involving acquisitions with and without an off‐resonance radiofrequency pulse targeting the macromolecule pool. This is a semi‐quantitative method, more biologically meaningful than magnetization transfer‐weighted imaging but very sequence dependent, meaning reproducibility and comparison between studies is limited, and influenced by T1 effects, inhomogeneities in the radiofrequency field and several other acquisition factors. Quantitative MT approaches aim to ameliorate these issues and provide a more biologically meaningful measure, acquiring multiple images and fitting a quantitative model to the data.

#### Pre‐processing

Pre‐processing is undertaken after image acquisition and before data analysis, aiming to correct for sources of imaging artifact that can alter the measured signal (Figure 3.1d–1f). This can reduce data quality, causing signal dropout, distortions and potential for erroneous conclusions. Motion is a key consideration in a movement disorder cohort, where there is potential for consistent between‐group differences. Subject motion can degrade results, potentially causing misalignment between images or slices or signal dropout. Taking steps to correct for this and to identify and remove outlying results reduces the potential for data bias.

Sources of artifact particularly relevant to dMRI relate to the echo planar imaging method, including B0 field inhomogeneities (where local regions in the imaging field are subject to different local magnetic environments), distorting signal and eddy currents (localized currents created by switching diffusion gradients rapidly). Amongst the dMRI studies, the majority undertook eddy current and motion correction with this either directly stated or inferred based on the analytical software employed. Whilst no studies detailed the specifics of the motion correction undertaken, most are likely to have corrected for between‐volume‐motion only, with many pre‐processing pipelines not accounting for within‐volume motion. Four studies additionally corrected for susceptibility distortions [45, 46, 50, 51]. Other sources of artifact particularly relevant to diffusion imaging, and not accounted for in any of the studies, include noise distribution bias (including thermal noise) related to the ‘noise floor’ (a measurable signal that remains in the absence of any true signal), gradient deviations due to nonlinearity of the diffusion gradient causing geometrical distortions, and Nyquist ghosts—an echo‐planar‐imaging‐specific artifact source where slight timing inconsistencies lead to the appearance of a ‘ghost’ image halfway across the main image.

The volumetric studies predominantly did not outline the pre‐processing steps to account for artifacts, stating that standard approaches were used. This would be presumed to include motion correction which is particularly key to enable accurate image registration and alignment for volumetric comparisons. Two studies undertook denoising [25, 71], and three inhomogeneity correction [25, 71, 72]. T1 relaxometry approaches are particularly influenced by non‐uniformity in the flip angle, and T2* relaxometry by magnetic field inhomogeneities; correction for these was only reported in one paper [47]. MTR imaging is particularly susceptible to radiofrequency field non‐uniformities, but only correction for motion was reported.

A number of additional potential artifacts can degrade data quality, and were not corrected for by any of the studies reviewed. These include signal drift, when the measured signal gradually changes over time (e.g. due to system heating), and Gibbs ringing, involving signal oscillations in regions with sharp boundaries in measured signal.

#### Analysis methods

A regional or whole brain focus can be taken during data analysis (Figure 3.2 gives example analysis approaches). The former involves targeted analysis of predefined locations/tracts, allowing a hypothesis‐driven approach, whilst a whole brain approach requires balancing the risk of false positive results (with inadequate multiple comparison correction) or false negative results (with more stringent correction), but does allow a more comprehensive analysis. Of the studies, 23 undertook a targeted, region‐based approach [12, 13, 16, 20, 21, 34, 35, 38, 39, 41, 42, 43, 48, 50, 51, 60, 62, 64, 65, 67, 69, 70, 73] and 28 a whole brain or whole grey/white matter‐based approach [17, 18, 19, 22, 23, 24, 25, 27, 28, 29, 31, 32, 44, 45, 54, 55, 57, 59, 63, 66, 68, 72, 74, 75, 76, 77, 78, 79]. The remaining 22 studies combined these approaches [9, 10, 11, 14, 15, 26, 30, 33, 36, 37, 40, 46, 47, 49, 52, 53, 56, 58, 61, 71, 80, 81], with five identifying the more specific regions post hoc using data from the whole brain analysis [9, 10, 11, 33, 53], an approach which has the potential to bias results.

Amongst the dMRI studies that took a regional approach, either this involved calculating properties of interest within the region of interest (ROI), as seen in 19 studies [9, 10, 12, 13, 14, 15, 33, 40, 41, 42, 43, 53, 56, 60, 61, 62, 65, 67, 70], or alternatively the ROIs can act as seed points to map white matter (WM) pathways using tractography (*n* = 18) [11, 30, 34, 35, 36, 37, 38, 39, 41, 46, 49, 50, 51, 53, 60, 61, 62, 64]. Untargeted approaches commonly employed included voxel‐based analysis (a voxel‐by‐voxel based approach looking at whole brain diffusion tensor measures) (*n* = 8) [9, 10, 11, 15, 37, 40, 61, 66]. This has been largely superseded by tract‐based spatial statistics (TBSS) (*n* = 14) [14, 30, 31, 32, 33, 44, 49, 52, 53, 54, 55, 56, 57, 58], overcoming many limitations surrounding misregistration. These studies all used standard measures such as FA, MD, AxD and RadD; these are useful but relatively non‐specific, potentially representing a range of biological or structural differences (Figure 2.2a). Of the remaining diffusion work, one study segmented into tissue compartments, calculating the average value within each [68], one assessed local diffusion homogeneity [57] and three used graph theory analysis, aiming to model whole brain connectivity [45, 59, 63].

Approaches used in the volumetric studies included volume measurements (using voxel‐based morphometry [VBM] or another volume approach) (*n* = 30) [15, 16, 17, 18, 19, 20, 21, 22, 23, 24, 26, 27, 28, 30, 33, 44, 46, 54, 55, 67, 69, 71, 73, 74, 76, 77, 78, 79, 80, 81], assessment of cortical thickness (*n* = 3) [29, 44, 57] or both (*n* = 6) [14, 25, 52, 58, 72, 75]. Differences in these measures are often attributed to hypertrophy or atrophy of associated brain regions, with inference of underlying pathological processes. However, image alignment and partial volume effects have particular propensity to influence these measures and therefore should be rigorously considered during analysis. For these studies, the segmentation process plays a vital role in the measured results. Manual segmentation has the potential for more anatomically accurate delineation of structures where anatomical boundaries may be unclear using automated methods; however, this introduces potential for inter‐rater variability, avoided with an automated approach. All papers used automated segmentation approaches, with one study undertaking additional manual thalamic segmentation.

Of the relaxometry studies, all three used a regional approach, with one additionally undertaking whole brain analysis [47], and the single MTR study employed a whole brain voxel‐by‐voxel comparison of the MTR with additional ROIs [15].

#### Correction for multiple comparisons

Imaging data analysis, particularly using whole brain approaches, can involve multiple comparisons over thousands of voxels, increasing the risk of type I errors. Commonly employed multiple comparison correction approaches identified in this review included the Bonferroni correction (*n* = 14) [16, 26, 29, 36, 37, 42, 43, 49, 51, 59, 62, 64, 68, 71], false discovery rate correction (*n* = 13) [11, 14, 17, 20, 22, 34, 40, 41, 50, 72, 77, 78, 79] and family wise error based correction (*n* = 19) [15, 21, 23, 27, 28, 31, 32, 33, 46, 54, 55, 58, 66, 67, 69, 70, 71, 75, 80, 81]. Several studies also analysed voxels as a cluster in the ROI, reducing the potential impact of multiple comparisons, either by setting cluster‐extent thresholds with *p* values within certain limits (*p* < 0.025 to *p* < 0.001, *n* = 12) [9, 10, 15, 25, 29, 30, 31, 33, 44, 60, 61, 67, 70, 73, 74, 76, 79], or by using threshold‐free cluster enhancement (*n* = 6) [14, 32, 33, 52, 58]. The remaining studies either undertook no documented multiple comparison correction (*n* = 11) [12, 13, 15, 30, 38, 48, 53, 55, 56, 57, 65], set a *p* value threshold (*n* = 3) [14, 18, 24] or did not state their approach (*n* = 2) [19, 35].

### Clinical characteristics and study findings

Seventy‐two of the human studies compared the dystonia cohort with an unaffected control group, matched for age and gender, with the remaining paper comparing genotypic and phenotypic dystonia subgroups [31] (Tables 2, 3, 4). There was substantial variation in cohort size, with ≤10 (*n* = 10) [24, 32, 34, 38, 40, 43, 49, 64, 65, 68], 10–20 (*n* = 25) [9, 10, 12, 14, 18, 19, 20, 29, 30, 35, 36, 37, 41, 42, 46, 47, 48, 50, 51, 56, 60, 67, 69, 75, 80], 20–30 (*n* = 15) [11, 15, 21, 26, 28, 33, 52, 57, 62, 70, 71, 74, 76, 79, 81] and >30 (*n* = 23) [13, 16, 17, 22, 23, 25, 27, 31, 39, 44, 45, 53, 54, 55, 58, 59, 61, 63, 66, 72, 73, 77, 78] participants.

#### Animal model imaging

These all involved DYT1 models, including knock‐out (KO) (*n* = 2) and knock‐in (KI) (*n* = 2) designs (Table 1). Two studies [82, 83] used an 11.1 T field strength, maximum *b* value 900 s/mm^2^ and 42 or unstated diffusion gradient directions; the others [84, 85] used a 9.4 T scanner with six directions and a maximum *b* value of 2138 s/mm^2^. KO models identified lower striatal free water and an associated higher MD [83], whilst higher FA was identified in the caudate, putamen, sensorimotor cortex and brainstem [85]. By contrast, elevated free water in the cerebellum and striatum [82] and lower FA in the superior cerebellar peduncle, sensorimotor cortex, caudate and putamen were identified in the KI forms [84].

**TABLE 1:** Animal models

First author, year	Dystonia type	Patients (M/F)	Controls (M/F)	Age, mean (Standard Deviation), patients/controls	Study aims	Methodology summary	Results
DeSimone, 2016 [82]	Mouse model DYT1 KI	20 (20/0)	20 (20/0)	Age range 3–6 months	Assessment of free water and free water corrected FA in DYT1 KI mouse model of dystonia	Whole brain resting state fMRI, free water and free water FA measurements	Elevated free water in cerebellum and striatum compared to wild type; no significant differences in free water corrected FA
DeSimone, 2017 [83]	Mouse model DTY1 *TorsinA* conditional KO	18 (10/8)	18 (7/11)	6.9 (0.8)/6.7 (1.3)	Assess for diffusion and functional differences	Diffusion and resting state MRI	↓ free water and ↑ MD in striatum
Vo, 2015 [85]	Animal model Tor1a heterozygous KO mice	25 (25/0)	25 (25/0)	—	Assess for metabolic and diffusion abnormalities in DYT1 mouse model	Ex vivo DTI and ^18^FDG PET	↑ FA in caudate, putamen, sensorimotor cortex and brainstem
Ulug, 2011 [84]	DYT1 knock‐in mice	8	6 (congenic)	16 weeks	Assess for PET and DTI differences	Micro‐PET, DTI and tractography (using DTI areas of abnormality as seed points)	DTI: ↓ FA (R) superior cerebellar tract, WM adjacent to (R) primary sensorimotor cortex, (L) caudate and putamen Tractography: ↓ streamline count between regions and ventral thalamus

Abbreviations: DTI, diffusion tensor imaging; F, female; FA, fractional anisotropy; ^18^FDG PET, ^18^F‐fluoro‐2‐deoxyglucose positron emission tomography; fMRI, functional magnetic resonance imaging; KI, knock‐in; KO, knock‐out; L, left; M, male; MD, mean diffusivity; R, right; WM, white matter.

**TABLE 2:** Genetic dystonia

First author, year	Dystonia type	Patients (M/F)	Control cohort and matching	Dystonia characterization	Study aims	Methodology summary	Results
Sako, 2015 [64]	DYT1	10 (4/6)	10 (age and gender matched, all right‐handed)	No blepharospasm or eye movement abnormalities	Assess for functional and structural differences in DYT1 cases versus controls related to visual motion perception	fMRI, deterministic tractography	↓ Streamline counts in ponto‐cerebellar projections
Vo, 2013 [68]	DYT1	7 (4/3)	8 (age and gender matched)	None detailed	To determine brain regions affected by dystonia	DTI with segmentation of tissue type	WM ↓ mean FA and ↑ mean diffusion
Argyelan, 2009 [60]	DYT1/6 MCs and NMCs	Total: 20 (10/10) (DYT1 7 MCs, 4 NMCs; DYT6 5 MCs, 4 NMCs)	8 (age and gender matched)	MCs and NMCs assessed, no other dystonia characterization	Determine the integrity of the cerebello‐thalamo‐cortical tract	Probabilistic tractography, comparison of MCs and NMCs	↓ Cerebello‐thalamo‐cortical streamline count in MCs and NMCs. NMC distal thalamo‐cortical and proximal cerebellar outflow abnormality
Cheng, 2012 [65]	DYT6	6 (4/2)	6 (no control age/gender data included but states no differences) Exclusions: other neurological/psychiatric diagnoses, neuroleptic medications and vascular risk factors	Reported: age of onset (and age at study), gene mutation, protein changes, motor phenotype	Assess FA and MD changes in motor pathways in DYT6 dystonia	ROI‐based assessment of DYT6 dystonia FA and MD values	↓ FA in sensorimotor area, ↑ MD in superior longitudinal fasciculus and supracapsular corticospinal tract
Carbon, 2008 [10]	DYT1/6 MCs and NMCs	Total: 15 (8/7) MCs: 7 (3/4) NMCs: 8 (5/3)	15 (age and gender matched controls; due to age difference between MCs and NMCs, each was compared to age‐matched controls not each other)	Reported: motor phenotype, duration of dystonia, BFMDRS score, medications	Assess motor pathway WM microstructure	MCs: whole brain FA Both cohorts: ROI‐based group difference analysis	MCs: ↓ FA in pons at base of left superior cerebellar peduncle and bilateral sensorimotor area; not seen in NMCs
Carbon, 2004 [9]	DYT1 MCs and NMCs	Total: 12 (7/5) MCs: 4 NMCs: 8	17 (age matched, not gender matched)	Reported: motor phenotype, medications	Assess WM motor pathways in DYT1 mutation carriers	Whole brain FA maps to identify areas for ROIs, plus prespecified ROI analysis	↓ FA in the subgyral WM of the sensorimotor cortex of DYT1 carriers (MCs and to a lesser extent NMCs)
Hanssen, 2018 [46]	DYT3	17 (17/0)	17 (age and gender matched)	Reported: disease duration, mean UPDRS‐III score, mean BFMDRS score, mean MoCA, mean FAB, mean TMT	Assess for volume changes in basal ganglia, cortex and cerebellum	VBM with ROIs in basal ganglia and sensorimotor cortex, whole brain analysis, separate cerebellum analysis; cortical thickness and subcortical volume; DTI analysis of WM pathways from areas of VBM abnormality	↓ Volume striatum and pallidum, ↓ volume frontal and temporal cortex
Hanssen, 2019 [69]	DYT3	18 (18/0)	19 (age and gender matched, other neurological disorders excluded)	Reported: BFMDRS, UPDRS‐III, MoCA‐P, disease duration	Assess for volume changes and iron accumulation (using T2* relaxometry) in striatum in DYT3	VBM and T2* relaxometry	↓ Volume and ↓ T2* relaxation rate in anteromedial putamen; ↓ T2* relaxation rates dorsolateral putamen
van der Meer, 2012 [67]	DYT11 (*SGCE*)	16 (8/8)	18 (age and gender matched)	Reported: BFMDRS, UMRS	Comparison of sensorimotor WM between DYT11 and controls	WM VBM and DTI	↑ FA and ↓ MD in subthalamic brainstem, ↓ MD in subgyral cortical sensorimotor areas. ↑ WM volume subthalamus brainstem
Beukers, 2011 [74]	DYT11	25 (12/13)	25 (age and gender matched, all right‐handed)	Reported: BFMDRS, UMRS, inheritance motor phenotype, psychiatric symptoms, medications Subanalyses excluding maternal inheritance	Assessment of grey matter volume	VBM	No significant difference between dystonia and controls; ↑ disease severity correlated with ↑ putamen volume
Blood, 2018 [36]	DYT12 (*ATP1A3*)	17 (16/1)	17 (age, gender and handedness matched)	Reported: duration of disease, BFMDRS‐M (total and speech), UPDRS‐III, HADS	Determine whether paralimbic‐striatal or sensorimotor connectivity is more greatly reduced compared to controls	Probabilistic tractography with group differences for paralimbic versus sensorimotor regions, TBSS	Tractography: ↑ caudate to paralimbic streamline count TBSS: widespread ↓ FA and ↑ MD in DYT12
Bruggemann, 2016 [32]	DYT12 (*ATP1A3*)	10 (10/0)	14 (age and gender matched, other neurological disorders excluded)	Reported: BFMDRS, UPDRS‐III	Assess WM tracts	TBSS in DYT12 compared to healthy controls	↑ MD apart from occipital lobes. ↓ FA in fornix, anterior thalamic radiation, corticospinal tract, superior corona radiata
Jochim, 2018 [49]	DYT27	4 (2/2)	12 (age and gender matched)	Reported: whether treated with botulinum toxin Standardized scanning at maximum botulinum toxin effect to limit movement artifact	Compare WM and fibre tract integrity	Whole brain TBSS analysis, tractography between predefined ROI pairs	TBSS: bilateral ↓ FA in cerebellar peduncles, pons, midbrain, thalamus, internal capsules and subcortical WM Tractography: significant ↓ FA in bilateral dentate to thalamus

Abbreviations: BFMDRS, Burke‐Fahn‐Marsden Dystonia rating scale; DTI, diffusion tensor imaging; F, female; FA, fractional anisotropy; FAB, frontal assessment battery; fMRI, functional magnetic resonance imaging; HADS, Hospital aniexty and depression scale; M, male; MC, manifesting carrier; MD, mean diffusivity; MoCA, Montreal Cognitive Assessment; NMC, non‐manifesting carrier; ROI, region of interest; TBSS, tract‐based spatial statistics; TMT, trail making test; UMRS, unified myoclonus rating scale; UPDRS‐III, Unified Parkinson's Disease rating scale; VBM, voxel‐based morphometry; WM, white matter.

**TABLE 3:** Idiopathic dystonia

Author, year	Dystonia type	Patients (M/F)	Control cohort matching	Dystonia characterization	Study aims	Methodology summary	Results
Sondergaard, 2021 [39]	Cervical dystonia	32 (3/29)	35 (age and gender matched, not neurological diagnoses, MoCA < 26, positive genetic testing for dystonia group)	Reported: TWSTRS, GDRS, tremor presence, disease duration, botulinum toxin treatment duration, medication use, psychiatric symptoms Scanning 3 months post botulinum toxin therapy	Assess for abnormalities in the dentatorubrothalamic tract	DTI	↓ FA (R) dentatorubrothalamic tract, ↓ MD and AxD in L
Pontillo, 2020 [21]	Cervical dystonia	27 (13/14)	27 (age and gender matched, excluded other neurological or psychiatric diagnoses)	Reported: symptom severity (Tui score), disease duration, botulinum toxin treatment duration Correlation of MRI measures with clinical features MRI undertaken during botulinum toxin wearing off phase	Assess cerebellar grey and white matter volume differences in cervical dystonia	ROI and voxel‐based cerebellar volume assessments	↓ GM volume anterior lobe and lobule VI; small clusters of ↓ GM volume (R) cerebellum, (L) midbrain, bilateral superior and middle cerebellar peduncles
Blood, 2019 [42]	Cervical dystonia	14 (4/10)	14 (age, gender and handedness matched, excluded other neurological or psychiatric diagnoses)	Reported: motor phenotype, symptom severity (Tsui score, BFMDRS, TWSTRS), disease duration, botulinum toxin treatment duration, medication use	Assessment of effects of botulinum toxin on WM FA asymmetry globus pallidus interna in dystonia	FA in ROI of WM medial to globus pallidus interna measured immediately prior to botulinum toxin and 4 weeks post	↓ FA asymmetry post botulinum toxin in cervical dystonia group, no significant difference between scans in controls
Gracien, 2019 [47]	Cervical dystonia	17 (8/9)	29 (age and gender matched)	Reported: symptom severity (Tsui scale) Scan undertaken at peak botulinum toxin effect	Assess for differences in quantitative MRI measures in cervical dystonia compared to controls	T1, T2, T2* and proton density maps; ROIs in basal ganglia, thalamus, WM, cerebellum and voxel‐wise whole brain	No significant differences
Delnooz, 2015 [76]	Cervical dystonia	23 (9/14)	22 (age and gender matched)	Reported: motor phenotype, symptom severity (TWSTRS), duration of symptoms, duration of botulinum toxin injections	Assessment of botulinum toxin effects on VBM	Whole brain VBM before and after botulinum toxin injection	Post botulinum toxin: ↑ GM volume (R) precentral sulcus
Aschermann, 2015 [48]	Cervical dystonia	12 (0/12)	12 (age and gender matched, no other major diseases)	Reported: symptom severity (TWSTRS), disease duration	Assessment of basal ganglia iron content	R2* relaxometry in thalamus, caudate, globus pallidus, putamen	↑ R2* in globus pallidus in cervical dystonia compared to controls
Prell, 2013 [15]	Cervical dystonia	24 (6/18)	24 (age and gender matched, no other neurological, psychiatric or vascular disease, dystonia group excluded dystonia elsewhere or family history)	Reported: UDRS, TWSTRS, MMSE, BDI, disease duration Last botulinum toxin injection 4 weeks pre MRI	Comparison of WM	VBM, DTI and magnetization transfer imaging	↑ FA: SMA, pons, thalamus, middle temporal and cingulate gyrus ↓ FA: precentral and postcentral gyrus, dorsolateral prefrontal cortex ↑ ADC: middle temporal gyrus, medial occipital lobe, somatosensory cortex *↑* Volume lentiform nucleus, (L) frontal eye field and bilateral medial occipital lobe; ↓ (L) precentral gyrus, SMA, medial temporal gyrus; (R) somatosensory association cortex *↑* Magnetization transfer ratio temporal and parietal lobes and (L) cingulate gyrus; ↓ primary and secondary visual cortices and (L) dorsolateral prefrontal and frontal cortex
Blood, 2012 [37]	Cervical dystonia	12 (6/6)	12 (1:1 matching for age, gender and handedness)	Reported: motor phenotype, symptom severity (Tsui score, BFMDRS), duration of dystonia, duration of prior botulinum toxin treatment, medications Scanned 1 week before botulinum toxin injection due	Determination of WM differences between brainstem and pallidum	Voxel‐wise FA and MD maps compared between groups. Probabilistic tractography	FA: ↓ (L) superior cerebellar peduncle WM; ↑ (L) substantia nigra Tractography: ↓ streamline count left ansa lenticularis to brainstem
Pantano, 2011 [20]	Cervical dystonia	19 (4/15); 12 had both scans	28 (age and gender matched, excluded brain abnormalities or neurological symptoms)	Reported: duration of dystonia, symptom severity (Tsui score) Scanned at time of maximal botulinum toxin treatment effect	Assess for GM volumes at baseline and 5 years later	VBM	Cervical versus HC at baseline: ↓ GM volume premotor and primary sensory cortices, (L) caudate head and putamen Cervical 5‐year follow‐up: (L) primary sensorimotor cortex volumes significantly reduced from baseline
Bonilha, 2009 [34]	Cervical dystonia	7 (1/6)	7 (1:1 age and gender matched)	Reported: motor phenotype	WM tract comparison between thalamus and MFG	Probabilistic tractography	Bilateral ↓ streamline count between thalamus and MFG
Bonilha, 2007 [40]	Cervical dystonia	7 (1/6)	10 (age and gender matched)	Reported: motor phenotype, treatments	Assess whether WM changes in DYT1 dystonia are also seen in idiopathic dystonia	(i) Voxel‐wise whole brain comparison of FA and MD, (ii) GM and WM FA and MD, (iii) ROI‐based analysis	↓ GM FA: posterior (R) thalamus ↓ WM FA: (R) thalamus and middle frontal gyrus ROI analysis: ↑ MD basal ganglia nuclei and connecting WM
Colosimo, 2005 [12]	Cervical dystonia	15 (4/11)	10 (states age and gender matched, no data provided; no other neurological conditions)	Reported: duration of dystonia, symptom severity (Tsui score), motor phenotype Scan at time of maximal botulinum toxin effect	Assessment for whole brain DTI differences	ROI‐based assessment of FA and MD	↑ FA bilateral putamen, ↓ FA corpus callosum ↓ MD (L) pallidum, (L) putamen, bilateral caudate
Draganski, 2003 [24]	Cervical dystonia	10 (3/7)	10 (age and gender matched)	Reported: motor phenotype, duration of symptoms, symptom severity (TWSTRS), duration of botulinum toxin Scanned at time of maximal botulinum toxin treatment effect	Assess for volume differences	VBM	↑ GM volume (R) globus pallidus interna, bilateral motor cortex and cerebellar flocullus; ↓ GM volume (R) SMA, visual cortex, dorsolateral prefrontal cortex
Merchant, 2020 [38]	Writer's cramp	9 (5/4)	15 (age and gender—no statistically significant differences, although 15‐year mean age difference) Other neurological or psychiatric disorders or use of opioid/cholinergic or GABAergic medications excluded	Reported: BFMDRS score, disease duration	Assess interactions between fine motor network nodes	DTI, fMRI and TMS	No significant difference in FA values in regions assessed
Berndt, 2018 [51]	Writer's cramp	18 (10/8)	18 (age and gender matched, all right‐handed)	Reported: symptom severity (ADDS, WCRS, writing movement score, writing speed), duration of dystonia, duration of botulinum toxin Last botulinum toxin >3 months prior to scan	Identify WM changes between premotor, cortical and subcortical regions	Probabilistic tractography—mean FA and CL	↓ FA between (L) MFG and putamen in patients versus controls
Zeuner, 2015 [81]	Writer's cramp	22 (9/13)	28 (age and gender matched, excluded other neurological or psychiatric conditions)	Reported: symptom severity (ADDS, WCRS) Last botulinum toxin >3 months prior to scan	Assess task‐related basal ganglia and cerebellar differences in writer's cramp compared to controls, and associated basal ganglia volume differences	VBM and fMRI	↑ GM volume in posterior putamen and globus pallidus
Delmaire, 2009 [11]	Writer's cramp	26 (9/17)	26 (age and gender matched, no other neurological conditions)	No botulinum toxin in last 6 months or other current medication	Identify WM changes between sensorimotor regions	Voxel‐based whole brain FA analysis and probabilistic tractography from areas of abnormality identified	↑ FA: posterior internal capsule, ventroposteriolateral thalamus Tractography: tracked to primary sensorimotor and brainstem ↓ Streamline counts near primary sensorimotor cortex Within tracts: ↑ FA (R) upper pons to corona radiata, (L) internal capsule to corona radiata
Battistella, 2018 [35]	Spasmodic dysphonia	12 (5/7)	12 (age and gender matched, all right‐handed, no neurological/psychiatric/ENT diagnoses)	54.1 (11)/55 (7.06)	Assess pathways between the insula and areas involved in auditory processing, motor preparation and output	Probabilistic tractography. Used the insula inputs to create subdivision to assess insula spatial organization	Probabilistic tractography: no significant differences Insula subdivisions: regions connecting with inferior frontal gyrus and primary motor cortex more ventro‐posterior in controls and more antero‐dorsal in dystonia
Kirke, 2017 [54]	Spasmodic dysphonia	Total: 40 With tremor: 20 (2/18) Without tremor: 20 (4/16)	20 (age and gender matched, all right‐handed, no neurological/psychiatric/ENT diagnoses)	Reported: disease duration, severity (voice breaks), motor phenotype Those on botulinum toxin >3 months post treatment when scanned	Assess for structural and functional changes compared to controls and abnormalities associated with tremor	fMRI, VBM and DTI	↑ FA in WM underlying the (L) inferior frontal gyrus. Corpus callosum differences with and without tremor ↑ GM volume (L) inferior frontal gyrus, bilateral putamen and (L) pallidum
Kostic, 2016 [14]	Spasmodic dysphonia	Adductor form: 13 (6/7)	30 (age and gender matched, right‐handed, no previous treatment with neuroleptics/anticholinergics, no other neurological, psychiatric, somatic, respiratory, swallowing or voice diagnoses)	Reported: symptom severity (BFMS, UDRS), disease duration, WM hyperintensity load, MMSE No botulinum toxin within 3 months preceding scan	Assess cortical morphology, basal ganglia and WM DTI	TBSS, cortical thickness, surface area and volume measures, basal ganglia DTI and volumetric measures	↑ MD and RD, and ↓ FA in corpus callosum, internal capsule, corticospinal/corticobulbar tracts, ↑ (R) caudate RD, AD, MD, ↑ (R) putamen RD and MD No cortical thickness differences Cortical surface area: ↑ primary somatosensory cortex, (R) primary motor cortex, (L) superior temporal gyrus, supramarginal and superior frontal gyri; ↓ Rolandic operculum and (L) superior parietal, supramarginal and lingual and (R) angular gyri
Simonyan, 2012 [25]	Spasmodic dysphonia	40 (15/25)	40 (age and gender matched, no other neurological, psychiatric, respiratory, voice or swallowing problem)	Reported: duration of dystonia, motor phenotype No botulinum toxin within 3 months preceding scan	Assess grey matter volume and cortical thickness abnormalities in spasmodic dysphonia	VBM and cortical thickness	↑ GM volume and cortical thickness in laryngeal sensorimotor cortex, inferior frontal gyri, superior and middle temporal gyri, supramarginal gyri ↓ Cortical thickness (L) anterior insula
Simonyan, 2008 [56]	Spasmodic dysphonia	Total: 20 (8/12) Adductor: 14 Abductor: 6	20 (age and gender matched, no other neurological, psychiatric or ENT conditions)	Reported: symptom severity (voice rates) and duration of dystonia and analysed for correlation between these and DTI scores	Assess for brain abnormalities	DTI (TBSS plus ROI based) and postmortem analysis of implicated regions	↓ FA: internal capsule. ↑ MD along corticobulbar and corticospinal tracts
Mantel, 2020 [50]	Embouchure dystonia	16 (14/2)	16 (age and gender matched, all right‐handed, excluded other neurological or psychiatric symptoms or signs, patient group no current treatments)	Reported: symptom severity (customized score and ADDS), disease duration, age started playing instrument, main instrument, daily training, total intracranial volume. Undertook linear regression analyses for clinical characteristics	Assess structural and functional abnormalities of cortical projections	Probabilistic tractography and seed‐based functional connectivity analysis	↓ AD between primary somatosensory cortex and putamen, ↑ between SMA and superior parietal cortex
Mantel, 2019 [71]	Embouchure dystonia	24 (21/3)	Healthy musicians: 23 Healthy non‐musicians: 24 (age and gender matched)	Reported: symptom severity (customized score), age started playing instrument, main instrument, daily training, total intracranial volume. Undertook linear regression analyses for clinical characteristics	Compare grey matter volume and symmetry in musicians with embouchure dystonia, healthy musicians and non‐musicians	Whole brain VBM and ROI volumes	Embouchure dystonia: ↑ GM volume primary sensorimotor cortex
Granert, 2011 [80]	Musician's dystonia (pianists)	11 (9/2)	Healthy pianists: 12 (age, gender and handedness matched)	Reported: symptom onset, fingers affected, age started playing, yearly playing time, yearly concerts	VBM assessment of putamenal GM volume differences in pianists with and without musician's dystonia	VBM in bilateral putamen	↑ GM volume (R) middle putamen in musician's dystonia
Guo, 2021 [59]	Blepharospasm	41 (17/24)	Hemifacial spasm controls: 41 HC: 41 (age and gender matched, excluded other neurological disorders, WM abnormalities on MRI, exposure to dystonia‐associated medications, anxiety symptoms)	Reported: education level, duration of disease, duration of botulinum toxin treatment, symptom severity (JRS), ADL impairment (BSDI). Correlation of clinical features with measures Scan at least 3 months following last botulinum toxin injection	Assess whether there are widespread structural network changes in blepharospasm compared to hemifacial spasm and healthy controls	Deterministic tractography, calculation of global and regional network measures	*↑* Global and local efficiency in both blepharospasm and hemifacial spasm, no difference between the two Additional hub regions: in primary head and face motor cortex and basal ganglia in blepharospasm compared to both control groups
Guo, 2020 [57]	Blepharospasm	29 (11/18)	30 (age and gender matched, all right‐handed, excluded other neurological disorders, drug and alcohol abuse, abnormalities on MRI, exposure to dystonia‐associated medications, anxiety symptoms)	Reported: disease duration, botulinum toxin duration, symptom severity (JRS), ADL impairment (BSDI). Correlation of imaging metrics with severity scores Scan at least 3 months following last botulinum toxin injection	Assess cortical thickness, FA and local diffusion homogeneity	Whole brain local diffusion homogeneity (LDH), FA and cortical thickness	*↑* LDH (L)—SLF, corpus callosum, posterior corona radiata, posterior thalamic radiation, cingulate bundle; (R)—SLF corona radiata, corpus callosum FA: no significant differences Cortical thickness: no significant differences
Hanganu, 2016 [29]	Blepharospasm	13 (4/9)	Hemifacial spasm control: 11 HC: 20 (age and gender matched, excluded neurological or psychiatric diagnoses and psychiatric, neurotropic and sedative medication use)	Reported: motor phenotype and symptom severity (UDRS, BRS, BDS, SRS) Scan when botulinum toxin dose due and 4 weeks later	Assess for GM microstructural differences in blepharospasm compared to hemifacial spasm and healthy controls, and for differences relating to timing of botulinum toxin	Cortical thickness analysis	Blepharospasm versus hemifacial spasm and HC: ↓ cortical thickness in frontorostral, supramarginal and temporal regions Blepharospasm post versus pre botulinum toxin: ↓cortical thickness in primary motor cortex and pre supplementary area
Horovitz, 2012 [30]	Blepharospasm	14 (0/14)	14 (age and gender matched)	Reported: symptom severity score (BFMDRS), duration of dystonia, duration of botulinum toxin treatment. Correlation of findings with age and dystonia severity	Assess for structural changes in blepharospasm	VBM and dMRI (TBSS and probabilistic tractography)	TBSS: no significant differences Tractography: ↓streamline count corticobulbar tract VBM: *↑* GM volume (L) lateral middle temporal gyrus, (R) postcentral gyrus, bilateral precuneus; ↓ GM volume (R) orbitofrontal cortex, (L) facial portion of primary motor cortex, (L) lateral inferior frontal gyrus, (R) occipital and anterior cingulate gyrus
Martino, 2011 [28]	Blepharospasm	25 (8/17)	24 (age and sex matched, all right‐handed, excluded other neurological disorders, secondary dystonias)	Reported: motor phenotype, disease duration, symptom severity (JRS, BFMDRS), geste antagoniste presence, botulinum treatment duration	Assess for grey matter volume differences	VBM	↑ GM volume (R) middle frontal gyrus, ↓ (L) primary somatosensory and superior temporal gyri
Suzuki, 2011 [22]	Blepharospasm	32 (10/22)	48 (age and gender matched, excluded other neurological or psychiatric diagnoses or secondary to medication cases)	Reported: symptom severity (JRS, activity index), duration of botulinum toxin treatment. Correlation of findings with disease duration and activity index	Assess grey matter density and correlation with disease duration and severity	VBM	↑ GM density in primary sensorimotor cortex and cingulate gyrus. Primary sensorimotor cortex density correlated with disease duration and severity
Etgen, 2006 [18]	Blepharospasm	16 (4/12)	16 (age and gender matched, all right‐handed, excluded secondary dystonias)	Reported: symptom severity (BDS, SRS), duration of dystonia, duration of botulinum toxin, time since last botulinum toxin treatment	Assess for GM volume differences in blepharospasm	VBM	↑ GM volume bilateral putamen; ↓ (L) inferior parietal lobule (correlated with duration of botulinum toxin)
Li, 2021 [77]	PKD	87 (71/16)	115 (age, education and gender matched, excluded history of substance/alcohol abuse, neurological, psychiatric or significant medical comorbidity, significant head motion on scanning)	Reported: education, disease duration, family history, attack frequency, affected side, treatments Antiepileptic drugs withdrawn 12 h prior to scan, and no treatment duration >3 months	Explore GM network topology and potential diagnostic value	VBM‐based morphological network matrices with support vector machine	Global differences: ↓ characteristic path length; ↑ local efficiency, clustering coefficient, normalized clustering coefficient, small‐worldness Use of GM morphological network matrices to classify cases 87.8% accuracy
Kim, 2015 [52]	PKD	25 (21/4)	25 (age and gender matched, all right‐handed, excluded chronic systemic, neurological or psychiatric comorbidities or alcohol/substance abuse)	Reported: age of onset, disease duration, family history, infantile convulsions, motor phenotype, treatment response Correlated findings with disease duration and family history	Assess for microstructural changes in PKD	Cortical thickness analysis and TBSS	↑ FA thalami and (R) anterior thalamic radiation Cortical thickness: no significant differences
Liu, 2020 [78]	Meige syndrome	46 (11/35)	65 (age and gender matched)	Reported: motor phenotype, symptom severity (BFMDRS), depressive symptoms (HAMD score), disease duration Comparison of those with and without depressive symptoms	Assess brain volume differences in Meige syndrome and associated depressive symptoms	VBM	Meige syndrome versus HC: ↓ GM volume (L) middle frontal orbital gyrus, temporal pole, insula; (R) temporal pole, precuneus, inferior parietal, inferior temporal and olfactory cortices With versus without depression: ↓ GM volume (L) cuneus and hippocampus; (R) angular gyrus, middle frontal gyrus, middle occipital gyrus
Tomic, 2020 [58]	Mixed TS (writer's cramp, laryngeal), NTSD (blepharospasm, cervical)	Total: 97 (33/64) (WC 21, SD 15, BSM 27, cervical 34)	83 (age and gender matched, all normal cognition excluded other neurological, psychiatric, laryngeal or ophthalmological conditions)	Reported: dystonia triggers, pain, sensory tricks, botulinum toxin treatment, symptom severity (UDRS, BFMDRS) Minimum 3 months after last botulinum toxin injection	Comparison of TSD versus NTSD	GM volumetric measures (VBM, cortical and subcortical measurements) and WM DTI TBSS	TBSS: more widespread WM changes in TSD versus NTSD VBM and cortical thickness: with FDR correction no significant differences; with *p* < 0.001 ↑ in TSD versus NTSD in primary sensory, (L) superior parietal, rostral middle frontal, supramarginal, fusiform, inferior temporal, (R) paracentral, precentral/premotor and inferior parietal gyri, basal ganglia, thalamus, hippocampus, amygdala NTSD versus control ↓ cerebellar volume. TSD volumes and cortical thickness ↑ in botulinum toxin treated
Hanekamp, 2020 [45]	Mixed TSD (WC, laryngeal)	LD: 17 (7/10) WC: 15 (6/9)	16 (age and gender matched, all right‐handed and normal cognition, excluded other neurological, psychiatric or laryngeal conditions)	Reported: age of onset, duration of dystonia, severity of dystonia (BFMDRS) Minimum 3 months since last botulinum toxin treatment, no oral medications	Assess for common and distinct differences in large scale structural network in writer's cramp and laryngeal dystonia	Graph theoretical analysis	Abnormal transfer of prefrontal and parietal nodes between neural communities and reorganization of normal hub architecture
Bianchi, 2019 [44]	Mixed TSD (WC, musician's focal hand dystonia; SD, singer's laryngeal dystonia)	Total: 47 TSD comparison: 16 (8/8) Laryngeal versus hand: 16 (8/8)/16 (8/8) Non‐musicians versus musicians: 16 (11/6)/16 (12/4)	16 (age and gender matched, excluded neurological, psychiatric and laryngeal conditions or cognitive impairment)	Reported: years of musical practice, dystonia duration, age of dystonia onset, instrument Minimum 3 months since last botulinum toxin treatment	Assess structural and functional differences in TSD	Resting state fMRI, VBM and TBSS	TSD versus HC: ↑ GM volume (R) premotor cortex, (L) inferior parietal lobule; ↓ FA (R) precuneus WM Laryngeal versus hand: ↑ volume (R) inferior frontal gyrus and insula and (L) superior parietal lobule; ↓ FA (L) middle temporal gyrus Musicians versus non musicians: ↑ volume (R) middle frontal gyrus, ↓ FA (R) superior longitudinal fasciculus, (R) corticospinal/corticobulbar tract (underlying laryngeal representation in precentral gyrus) and (L) corticospinal tract (underlying hand representation in precentral gyrus)
Berman, 2018 [70]	Mixed CD and blepharospasm	Total: 30 (8/22) BSM: 12 (4/14) Cervical: 18 (4/8)	30 (age and gender matched, excluded secondary or childhood onset dystonias, other neurological examination findings)	Reported: dystonia severity (JRS, TWSTRS) Minimum 10 weeks since last botulinum toxin treatment Correlation analyses of severity with imaging findings	Assess for microstructural differences between patients with BSM and CD	Whole brain and ROI comparison of FA and MD values	CD versus controls: ↓ FA (R) cerebellum, ↑ MD (L) caudate CD versus BSM: ↓ FA (R) cerebellum Bilateral caudate. BSM: ↓ FA (R) globus pallidus internus and (L) red nucleus compared to controls and cervical dystonia
Vilany, 2017 [72]	Mixed blepharospasm, CD and oromandibular dystonia	49 (15/34)	79 (gender matched, one subgroup significant age difference from controls, excluded neurological and psychiatric comorbidities, HC normal neurological examination)	Reported: age of symptom onset, symptom severity (BFMDRS), duration of botulinum toxin treatment	Assess cortical thickness and subcortical volume in craniocervical dystonia	Cortical thickness and subcortical volume	Dystonia versus controls: ↓ cortical thickness (R) precuneus and lateral occipital gyrus
Waugh, 2016 [26]	Mixed CD, SD	CD: 17 SD: 7	CD controls: 17 SD controls: 7 (1:1 matched for age, gender and handedness)	Reported: symptom severity (Tsui score, V‐RQOL), timing of most recent botulinum toxin	Compare regional volumes in focal dystonia and healthy controls	Volumetric measure: 8 automated ROIs in motor control regions; manual ROI thalami; VBM	↓ Thalamic volume
Pinheiro, 2015 [53]	Mixed CD, blepharospasm, oromandibular	Total: 40 (12/28) CD: 18 BSM: 5 BSM and OMD: 9 Cervical, BSM, OMD: 8	40 (age and gender matched, excluded other neurological examination abnormalities, secondary dystonias)	Reported: symptom severity (BFDRS), disease duration, botulinum toxin treatment duration CD: 52 (12.2) BSM: 61.8 (10.3) BSM, OMD: 68.5 (5) CD, BSM, OMD: 63.1 (11.1) HC: 56 (15)	Evaluate WM microstructure	TBSS, tractography and ROI‐based comparison	No significant differences
Piccinin, 2015 [79]	Mixed CD, blepharospasm, oromandibular	27 (9/18)	54 (age and gender matched, HC normal neurological examination)	Reported: disease duration, botulinum toxin treatment duration, symptom severity (BFMDRS), motor phenotype Clinical correlation of imaging findings	Assess for GM volume differences in craniocervical dystonia compared to controls	VBM	Dystonia versus controls: none remained significant with FDR correction With *p* < 0.001: ↓ GM volume cerebellar vermis IV/V, superior frontal gyri, precuneus, anterior cingulate, paracingulate, insula, lingual gyrus, calcarine fissure; (L) SMA, inferior frontal gyrus, inferior parietal gyrus, temporal pole, supramarginal gyrus, Rolandic operculum, hippocampus, middle occipital gyrus, cerebellar lobules IV/V, superior and middle temporal gyri; (R) middle cingulate and precentral gyrus
Yang, 2014 [66]	Mixed blepharospasm and oromandibular	Total: 31 (8/23) BSM: 20 (5/15) BSM and OMD: 11 (3/8)	11 (age and gender matched, secondary dystonias excluded, neurological and psychiatric comorbidities excluded)	Reported: dystonia severity (JRS, BFMDRS), presence of sensory trick No medications in 24 h prior to scan Correlation of imaging findings with clinical parameters	Compare diffusion measures between blepharospasm and blepharospasm with oromandibular dystonia with healthy controls	Whole brain voxel‐based analysis of DTI metrics	Blepharospasm: ↓ FA (L) anterior cerebellum; ↑ MD and RadD (R) lentiform and thalamus Blepharospasm with oromandibular dystonia: ↓ FA (R) precuneus of parietal lobe, ↑MD (R) lentiform nucleus and insula, ↑ AxD (R) insula
Ramdhani, 2014 [55]	Mixed TSD (writer's cramp, laryngeal) and NTSD (CD, blepharospasm)	Total: 45 (14/31) WC: 12 (8/4) SD: 12 (6/5) Cervical: 11 (7/4) BSM: 10 (9/1)	24 (age and gender matched, all right‐handed, excluded other neurological, psychiatric or laryngeal problems)	Reported: disease duration Minimum 3 months since last botulinum toxin treatment	Comparison of microstructural properties between TSD and NTSD	VBM and DTI	NTSD versus HC: ↓ *FA*: corpus callosum, internal capsule, (R) primary sensorimotor cortex; *↑ FA*: corpus callosum, cingulate gyri, occipital cortex, midbrain/pons, cerebellum; *↑* GM volume (L) primary sensorimotor cortex, middle temporal gyrus, thalamus, cerebellum; ↓ GM volume (R) middle frontal gyrus, inferior temporal gyrus, occipital cortex, putamen and ventral striatum TSD versus HC: ↓ *FA*: frontal gyrus, (R) anterior internal capsule, corpus callosum; *↑ FA*: cerebellum; *↑* GM volume (L) premotor cortex, inferior parietal lobule, cerebellum; (R) primary sensorimotor cortex, superior temporal and supramarginal gyri, middle and inferior temporal gyri NTSD versus TSD: ↓ FA in TSD—frontal gyrus, corpus callosum, putamen, (R) premotor cortex, internal capsule; ↓ FA in NTSD—WM underlying middle cingulate gyrus, (L) primary sensorimotor cortex, (L) inferior parietal lobule; *↑* GM volume (L) cerebellum specific to NTSD; *↑* GM volume middle frontal gyrus, middle/posterior cingulate cortex, inferior temporal gyrus, putamen, caudate, striatum, cerebellum; (R) primary somatosensory cortex, superior temporal gyrus, and occipital cortex specific to TSD
Piccinin, 2014 [73]	Mixed CD, blepharospasm, oromandibular	35 (gender mix not stated)	35 (age and gender matched although gender mix not stated, neurological comorbidities excluded)	Reported: motor phenotype, dystonia severity (BFMDRS), duration of botulinum toxin treatment Correlation of clinical features with imaging measures	Assess for cerebellar volume differences	Manual volumetry	↑ GM volume (L) I–IV cerebellar lobules; ↓ GM volume left lobule VI and crus I and (R) VI, VIIIb and crus I ↑ GM volume brainstem (especially pons)
Cerasa, 2014 [75]	Mixed (dystonic tremor—neck and limb)	12 (6/6)	Essential tremor: 14 HC: 23 (age and gender matched, excluded brain lesions, botulinum toxin treatment, depressive symptoms, other neurological or psychiatric disease)	Reported: tremor severity (FTM or UDRS rating scales), disease duration, family history, MMSE	Comparison of morphology in dystonic tremor and essential tremor	VBM and cortical thickness	Dystonic tremor: (L) sensorimotor cortex thickening and ↑ volume
Fabbrini, 2008 [13]	CD and blepharospasm	Total: 34 (13/21) BSM: 16 (7/9) Cervical: 18 (6/12)	BSM HC: 16 CD HC: 16 (age and gender matched, excluded other neurological comorbidities and MRI abnormalities)	Reported: motor phenotype, symptom severity (Tsui/BSS) assessed immediately prior to botulinum toxin therapy, duration of botulinum toxin therapy Scanning undertaken at point of maximum botulinum toxin efficacy	Assess whether previous CD DTI findings were reproducible and specific to CD	DTI ROI‐based comparison of CD, BSM and HC	CD: ↓ FA corpus callosum, ↑FA in bilateral putamen compared to HC; ↓ MD (L) caudate and (R) putamen and ↑ MD in bilateral prefrontal cortex and (L) SMA
Obermann, 2007 [19]	Mixed CD and blepharospasm	Blepharospasm: 11 (4/7) Cervical: 9 (2/7)	14 (age, gender and handedness matched, excluded other neurological, psychiatric or somatic comorbidities)	Reported: dystonia duration, duration dose and interval of botulinum toxin treatment	Assess for grey matter volume differences in cervical dystonia and blepharospasm	VBM	Cervical: ↑ GM volume thalamus and caudate, superior temporal lobe, (L) cerebellum; ↓ GM volume putamen Blepharospasm: ↑ GM volume caudate and cerebellum; ↓ GM volume putamen and thalamus
Blood, 2006 [43]	Mixed (CD, WC)	6 (3/3)	6 (age, gender and handedness matched)	Reported: motor phenotype, symptom severity pre and post botulinum toxin (Tsui/BFMDRS)	Assessment of effects of botulinum toxin on FA values	Scanned once prior to botulinum toxin and once 4 weeks post Bilateral ROIs to measure FA in WM between putamen/pallidum and thalamus	FA asymmetry in patients pre botulinum toxin not seen in post treatment or HC scans
Garraux, 2004 [27]	Focal hand dystonia (WC and musician's)	36 (21/15) writer's cramp: 31 musician's: 5	36 (age, gender and handedness matched, excluded head trauma, epilepsy, brain surgery, systemic illness, drug or alcohol misuse)	Reported: duration of dystonia, hand affected, dystonia severity (BFMDRS, timed writing test)	Assess for VBM differences in focal hand dystonia compared to controls	VBM	↑ GM volume perirolandic hand area

Abbreviations: ADC, apparent diffusion coefficient; ADDS, Arm dystonia disability scale; ADL, activities of daily living; AxD, axial diffusivity; BDI, Beck's Depression Inventory; BDS, Blepharospasm disability index; BFMDRS, Burke‐Fahn‐Marsden Dystonia Rating scale; BRS, blepharospasm rating scale; BSDI, blepharosmasm disability index; BSM, blepharospasm; BSS, blepharospasm severity scale; CD, cervical dystonia; CL, linear anisotropy; dMRI, diffusion magnetic resonance imaging; DTI, diffusion tensor imaging; ENT, ear nose and throat; F, female; FA, fractional anisotropy; FDR, false discovery rate; fMRI, functional magnetic resonance imaging; FTM, Fahn‐Tolosa‐Marin rating scale; GABA, gamma aminobutyric acid; GM, grey matter; GDRS, Global Dystonia rating scale; HAMD, Hamilton Depression rating scale; HC, Healthy Control; JRS, Jankovic rating scale; L, left; LD, laryngeal dystonia; LDH, local diffusion homogeneity; M, male; MD, mean diffusivity; MFG, middle frontal gyrus; MMSE, mini mental state examination; MoCA, Montreal Cognitive Assessment; MRI, magnetic resonance imaging; NTSD, non‐task‐specific dystonia; OMD, oromandibular dystonia; PKD, paroxysmal kinesigenic dyskinesia; R, right; RadD, radial diffusivity; ROI, region of interest; SLF, superior longitudinal fasciculus; SMA, supplementary motor area; SRS, severity rating scale; TBSS, tract‐based spatial statistics; TMS, transcranial magntic stimlation; TSD, task‐specific dystonia; TWSTRS, Toronto western spasmodic torticollis rating scale; UDRS, unified dystonia rating scale; VBM, voxel‐based morphometry; V‐RQOL, voice related quality of life; WC, writers cramp; WCRS, writers cramp rating scale; WM, white matter.

**TABLE 4:** Mixed genetic and idiopathic dystonia cohorts

Author, year	Dystonia type	Patients (M/F)	Control cohort matching	Dystonia characterization	Study aims	Methodology summary	Results
Li, 2020 [63]	PKD	Total: 78 (63/14) PRRT2 carriers: 35 PRRT2 non carriers: 43	40 (age and gender matched) Excluded other neurological, psychiatric or serious physical conditions, drug and alcohol excess	Reported: education, disease duration, age of onset, family history, motor phenotype, treatments and duration	Comparison of topological properties of WM structural connectome	Genetic sequencing and graph theory and network‐based statistical approaches	↓ Local efficiency; PRTT2 had longer characteristic path length and ↓ global efficiency; ↓ nodal characteristics in left thalamus and inferior frontal gyrus; PRRT2 carriers also left fusiform and bilateral middle temporal gyrus
Li, 2019 [33]	PKD	Total: 30 (27/3) PRRT2 carriers: 15 PRRT2 non‐carriers: 15	15 (age and gender matched, all right‐handed)	Reported: age of onset, disease duration, motor phenotype, gene mutation, treatment	Assess relationship in PKD between gene mutations, structural imaging changes and clinical symptoms	Genetic sequencing, TBSS, VBM and ROI (based on TBSS and VBM abnormalities)	↓ MD left corticospinal tract and anterior internal capsule. No genotypic differences. Disease duration negatively correlated with MD, RadD and AxD ↓ GM volume in pre SMA and inferior frontal gyrus
Long, 2017 [41]	PKD	Total: 20 (14/6) PRRT2 carriers: 8 (6/2) PRRT2 non‐carriers: 12 (8/4)	20 (age and gender matched) Excluded drug and alcohol use, other neurological, psychiatric or serious physical condition	Reported: age of onset, disease duration, motor phenotype, treatment and duration	Assessment of functional and structural connectivity between thalamus and cortex	fMRI and DTI between thalamus and cortical regions	↑ AD between ventral lateral/anterior nucleus and motor/premotor cortex in PKD. ↑ FA and AxD in mediodorsal nucleus and ventral lateral/anterior nuclei
Bianchi, 2017 [31]	Spasmodic dysphonia	Total: 89 (11/72) Sporadic adductor 30; abductor 30 Familial adductor 22; abductor 7	No healthy control group (matched between groups for age, gender and handedness) Excluded other neurological, laryngeal or psychiatric conditions	Reported: age of onset, disease duration Minimum 3 months since last botulinum toxin treatment	Assess for phenotype‐ and genotype‐related differences in FA values	Whole brain TBSS performed, with comparison of phenotype and genotype groups	↓ FA: adductor versus abductor: right superior corona radiata and splenium of corpus callosum Sporadic versus familial: right sagittal stratum (adductor only), left superior longitudinal fasciculus (adductor and combined)
Fujita, 2018 [61]	MC and NMC DYT1 and DYT6, and idiopathic	Total: 40 (17/23) MC DYT1: 10 MC DYT6: 6 NMC DYT1: 2 NMC DYT6: 9 Sporadic: 13	12 (age and gender matched)	Reported: motor phenotype (BFMDRS)	Assess WM tracts between visual motion perception related pattern nodes	fMRI in visual motion perception; DTI used to assess pathways between fMRI nodes	↓ Fibre counts between identified visual motion perception related pattern nodes
Draganski, 2009 [23]	MC and NMC DYT1 and idiopathic	Total: 51 MC DYT1: 11 (3/8) NMC DYT1: 11 (5/6) Idiopathic with family history: 15 (5/10) Idiopathic without family history: 14 (8/6)	28 (age and gender matched) Excluded other neurological conditions, surgical dystonia treatments	Reported: disease duration, site of onset, motor phenotype (BFMDRS), medication Minimum 4 months post last botulinum toxin	Assess effect of genotype and phenotype on structure	VBM	NMC DYT1 and idiopathic ↑ putamen volume versus both HC and MC DYT1 Idiopathic ↑ putamen and pallidum volume versus HC NMC ↑ sensorimotor cortex volume versus HC In MC DYT1, ↓ putamen volume correlated with ↑ symptom severity
Vo, 2015 [62]	DYT1, DYT6 and idiopathic	Total: 25 (11/14) DYT1: 10 DYT6: 3 Idiopathic: 12 Limb subtype: 7 leg involved, 18 not involved	8 (age and gender matched) Excluded other neurological conditions	Reported: motor phenotype (BFMDRS) Minimum 3 months post last botulinum toxin	Assess for DTI changes associated with specific limb manifestations	Assessing subcortical WM, using fMRI to identify limb‐specific motor cortex, genotype and phenotype specific	↓ FA and ↓ fibre count in WM deep to leg area without leg dystonia and deep to hand area without arm dystonia. Genetic dystonia ↓ fibre counts in cerebello‐thalamic segment of tract
Bai, 2021 [16]	Mixed	50 (23/27)	50 (age and gender matched, control group other neurosurgical presentations excluding basal ganglia pathology, cerebrovascular abnormalities, inflammatory conditions, hydrocephalus)	Reported: age of onset, disease duration, genotype	Assessment of basal ganglia volume and T2 hyperintensities in mixed primary dystonia cohort planned for deep brain stimulation compared to controls with other neurosurgical diagnoses	Basal ganglia T2 hyperintensity and volume assessment using Brainlab navigation software	↑ Globus pallidus and ↓ caudate and putamen volumes compared to controls
Egger, 2007 [17]	Mixed (generalized, cervical, hand)	Generalized: 9 (6/3) Cervical: 11 (6/5) Hand: 11 (8/3)	31 (age and gender matched) Excluded head trauma, epilepsy, brain surgery, systemic illness, drug/alcohol excess	Reported: disease duration, motor phenotype (BFMDRS/Tsui score)	Compare brain volume between dystonia subtypes and between dystonia and HC	Whole brain VBM	Dystonia versus HC: ↑ GM volume bilateral globus pallidus interna, nucleus accumbens and prefrontal cortex; left inferior parietal lobe

Abbreviations: AxD, axial diffusivity; BFMDRS, Burke Fahn Marsden Dystonia rating scale; DTI, diffusion tensor imaging; FA, fractional anisotropy; fMRI, functional magnetic resonance imaging; GM, grey matter; HC, Healthy control; MC, manifesting carrier; MD, mean diffusivity; NMC, non‐manifesting carrier; PKD, paroxysmal kinesigenic dyskinesia; RadD, radial diffusivity; ROI, region of interest; SMA, supplementary motor area; TBSS, tract‐based spatial statistics; VBM, voxel‐based morphometry; WM, white matter.

#### Genetic dystonia

Thirteen studies explored genetically homogeneous cohorts, involving DYT1 (*TorsinA* mutation) (*n* = 3) and DYT6 (*THAP1* mutation) (*n* = 1, or both *n* = 2), DYT3 (*n* = 2), X‐linked dystonia parkinsonism (DYT12, *ATP1A3* mutation, *n* = 2), myoclonus dystonia (DYT11, *SGCE* mutation, *n* = 2) and DYT27 (*n* = 1).

Amongst the DYT1 and DYT6 studies, a lower FA was demonstrated in the WM deep to the sensorimotor cortex and in cerebellar projections, with fewer tractography streamlines in the cerebellar outflow tracts in patients compared to controls [9, 10, 60, 64, 65]. Other findings included higher MD in the superior longitudinal fasciculus and supracapsular corticospinal tract [65]. Comparisons of manifesting and non‐manifesting gene mutation carriers (MC and NMC respectively) found intermediate WM FA and tractography changes for NMC in the cerebellar outflow region and sensorimotor cortex [9, 60]. The DYT3 studies implicated a role for the basal ganglia with evidence of reduced overall volume and a slower T2* relaxation rate in the putamen [46, 69].

The two studies involving DYT12 cohorts revealed widespread elevated MD on TBSS analysis, one with corresponding lower FA [36] and the other lower FA in the fornix, anterior thalamic radiation, corticospinal tract and superior corona radiata [32]. Use of more targeted probabilistic tractography between the paralimbic/sensorimotor cortex and caudate/putamen found increased fibre counts [36]. A single diffusion study of S*GCE*‐mutation‐positive patients (DYT11) found higher FA and lower MD in the subthalamic brainstem and subgyral sensorimotor cortex respectively, as well as an increased subthalamic WM volume [67]; a further study of grey matter (GM) volume identified no differences, although higher disease severity was associated with greater putaminal volume [74]. For those with DYT27 mutations, a single TBSS study found lower FA in the cerebellar peduncles, pons, midbrain, cerebral peduncles, thalamus, internal capsule, and frontal and parietal WM, with more targeted predefined tractography between thalamus/putamen and cortex revealing lower FA between the dentate nucleus and thalamus [49].

#### Idiopathic dystonia

Fifty‐one studies were identified, including cervical dystonia (*n* = 12), writer's cramp (*n* = 4), spasmodic dysphonia (*n* = 6), embouchure dystonia (*n* = 2), paroxysmal kinesigenic dystonia (*n* = 2), blepharospasm (*n* = 7), musician's hand dystonia (*n* = 1) and Meige syndrome (*n* = 1), with a further 15 studies combining multiple forms.

##### Task‐specific focal dystonias

Diffusion studies involving individuals with writer's cramp have found conflicting results. One identified no FA differences between patients and controls [38], whereas another noted lower FA in the tracts between the middle frontal gyrus and putamen [51]. Conversely, higher FA values have been noted in multiple regions, including the posterior internal capsule and ventroposteriolateral nucleus of the thalamus, which on tractography corresponded to areas tracking to either the primary sensorimotor cortex or brainstem [11], and higher volumes in the posterior putamen and globus pallidus [81].

Of the studies investigating spasmodic dysphonia, lower FA and higher MD were identified in the right internal capsule in the patient group using TBSS, with higher MD in the corona radiata, internal capsule, thalamus, cerebral peduncle and cerebellum [14, 56]. Language regions, namely the inferior frontal gyrus, were also implicated, with corpus callosal differences associated with the presence or absence of a tremor. Others have found a lower FA and higher MD and RadD in the corpus callosum and WM tracts, with higher AxD in the more anterior WM regions [14]. Another study used more targeted probabilistic tractography focused on regions involved in speech between the insula and cortex, noting no significant differences between patients and controls [35]. Volumetric studies have shown some overlap in findings with increased cortical surface area and GM thickness in the inferior frontal gyrus and primary sensory and motor cortices [14, 25] and, on occasion, implication of the superior and middle temporal gyri, superior frontal gyrus, putamen and pallidum [54].

Studies focused on musician's dystonia included embouchure dystonia, noting lower AxD between the primary somatosensory cortex and putamen and higher AxD between the supplementary motor area (SMA) and the superior parietal cortex [50]. VBM volumetric studies have identified greater sensorimotor cortex and putaminal GM volume compared to both unaffected musicians and non‐musicians [71].

##### Non‐task‐specific focal forms of dystonia

Amongst cervical dystonia cohorts lower WM FA has been identified compared to controls in regions including the superior cerebellar peduncles, thalamus, middle frontal gyrus, corpus callosum, and prefrontal and visual cortices [34, 37, 39, 40]. By contrast, higher WM FA has been observed in the substantia nigra [37], putamen [12], pons, thalamus, supplementary motor cortex, middle temporal gyrus and cingulate gyrus [15]. Probabilistic tractography studies have corroborated abnormalities relating to thalamic projections, including lower fibre counts between the thalamus, middle frontal gyrus and brainstem [34]. MD value differences have been conflicting, with higher values reported in the basal ganglia and cerebello‐thalamo pathways [39, 40], whilst others have reported lower values in the caudate, pallidum and putamen [12]. Volumetric studies have yielded similarly contrasting results with some identifying larger [24], and others smaller, volumes in the caudate, putamen, globus pallidus and primary motor cortex [15], primary sensory cortex, premotor cortex, SMA, medial temporal gyrus and prefrontal cortex [20]. Specific focus on the cerebellum found smaller GM volumes in the anterior and VI lobules and smaller cerebellar peduncles [21]. A single longitudinal study identified a reduction in left primary sensorimotor cortex volumes over 5 years [20]. Finally, no significant differences were observed using a T1, T2, T2* relaxometry approach and proton density maps [47], whereas others identified higher R2* values in the globus pallidus [48], potentially implicating increased brain iron deposition.

Studies of blepharospasm have not identified FA differences [30, 57] but did note reduced average tract volumes and streamline counts between the brainstem and motor cortex [30], and increases in local diffusion homogeneity in multiple regions, correlating with disease severity [57]. Volumetric studies have again produced mixed findings including higher GM volume in the putamen, cingulate and middle frontal gyrus, lower orbitofrontal and occipital cortical volumes, variable primary sensorimotor cortex volumes, and lower cortical thickness in frontal and temporal regions [18, 22, 28, 30].

Studies examining idiopathic paroxysmal kinesigenic dyskinesia (PKD) have found higher FA in the thalami and right anterior thalamic projections [52], with no cortical thickness differences compared to controls. Using VBM‐based morphological network matrices, global differences were noted, including shorter path length and higher local efficiency in the clinically affected cohort [77].

##### Mixed idiopathic cohort studies

A number of studies have compared task‐ and non‐task‐specific dystonias, identifying lower FA in the middle/inferior frontal gyrus, corpus callosum, putamen and premotor cortex in task‐specific forms, and in the middle cingulate gyrus and primary sensorimotor cortex in non‐task‐specific forms [55, 58]. Volumetric GM comparisons have found widespread higher GM volume and cortical thickness in task‐specific forms, including sensory and premotor cortex, parietal and temporal regions, basal ganglia and thalamus, whilst cerebellar measurements appear to vary [55, 58].

Several studies have collectively assessed cranio‐cervical dystonias (cervical, blepharospasm and oromandibular). In combination, one study found no consistent differences compared to unaffected controls [53], whilst analysis of the individual forms identified both higher [70] and lower [13] MD in key motor regions in cervical dystonia, and lower FA and higher MD in the basal ganglia in blepharospasm [66, 70]. Volumetric studies have identified higher volumes in the caudate and lower volumes in the putamen in both cervical dystonia and blepharospasm, with a higher thalamic GM volume in cervical dystonia and lower in blepharospasm [19]. Others have identified increased cerebellar GM volumes, reduced cortical thickness and an overall tendency towards smaller GM volumes [72, 73, 86].

Other comparisons have included (i) a mixed group of cervical and laryngeal dystonia, identifying lower thalamic volumes compared to healthy controls [26], (ii) laryngeal dystonia in musicians and non‐musicians demonstrating lower FA in the musicians in the superior longitudinal fasciculus, corticospinal and corticobulbar tracts [44], (iii) dystonia with and without tremor, where larger volumes and increased cortical thickness in the sensorimotor cortex were observed in those with tremor [75], and (iv) combined writer's cramp and musician's hand dystonia identifying larger volumes in the sensorimotor cortex compared to controls [27].

#### Mixed genetic and idiopathic cohort studies

Three studies involved PKD cohorts, with and without *PRRT2* mutations, with a lower MD identified in the corticospinal tracts and anterior internal capsule of the PKD group, negatively correlating with disease duration, and a higher FA in the thalamic nuclei and premotor/motor cortex [33, 41, 63]. One study additionally assessed for volumetric differences, finding lower GM volumes in the pre‐SMA and inferior frontal gyri in the PKD group [33]. Combined cohorts of DYT1, DYT6 and idiopathic dystonia have suggested pathways involved in visual motion [61], limb‐specific lower FA in the WM deep to the hand or leg areas of the motor cortex [62], and genotype‐specific tractography differences with fewer cerebello‐thalamic tract streamlines in inherited forms of dystonia [62]. Spasmodic dysphonia likewise demonstrated both phenotype‐ and genotype‐specific patterns, with abductor compared to adductor forms having lower FA in the corona radiata and corpus callosum, and familial compared to sporadic forms identifying lower FA in the striatum and higher FA in the left superior longitudinal fasciculus [31]. Volumetric studies in mixed cohorts have identified higher GM volume in the globus pallidus, prefrontal cortex and parietal lobe, together with lower volumes in the caudate and putamen [16, 17].

## DISCUSSION

**FIGURE 4:**
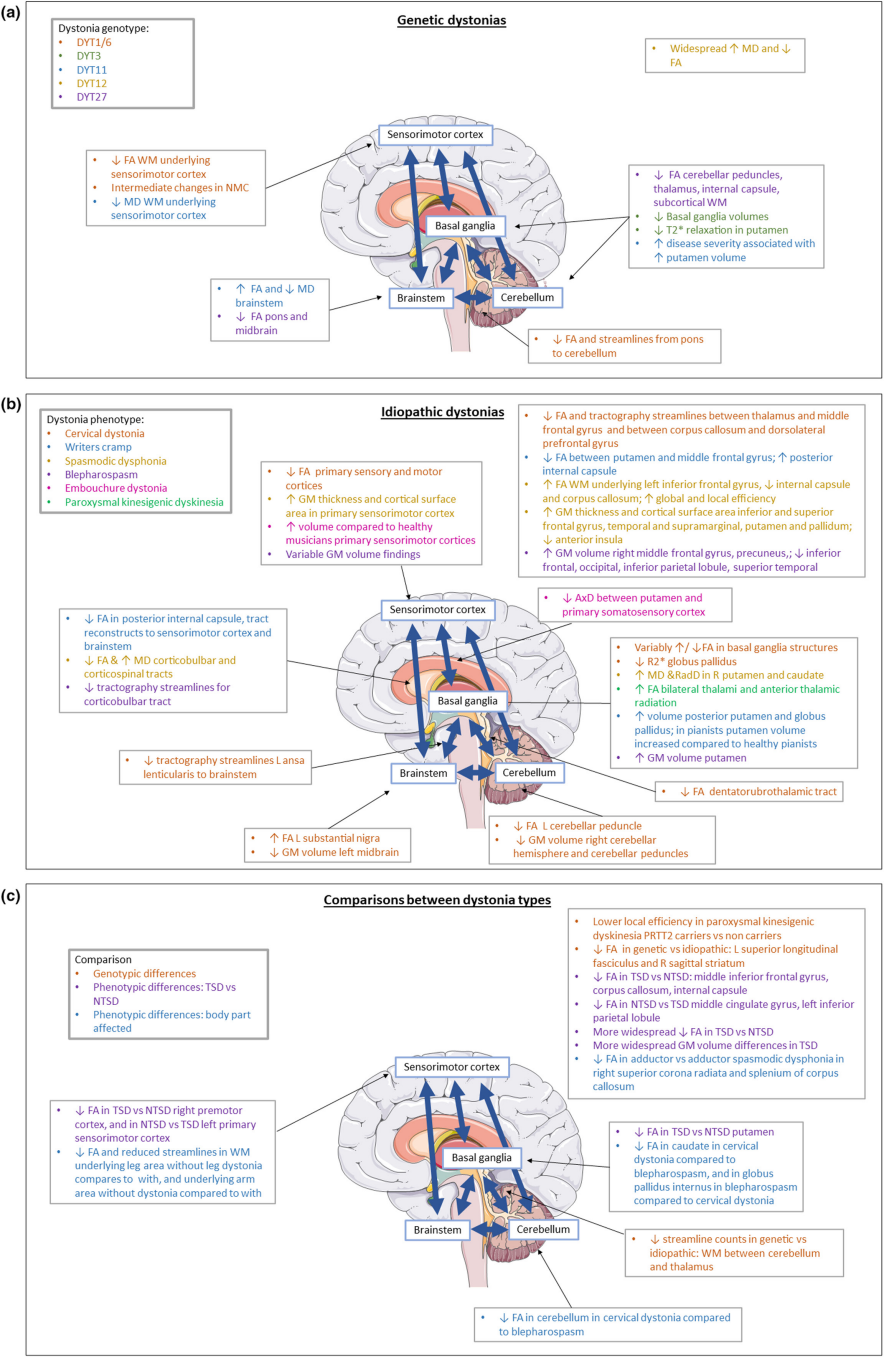
Summary of structural MRI findings in dystonia. (a) shows findings amongst genetic dystonias (b) amongst idiopathic dystonias and part (c) shows comparisons of different dystonia subtypes. Values are relative to healthy controls unless otherwise stated. FA, fractional anisotropy; MD, mean diffusivity; GM, grey matter; WM, white matter; L, left; R, right; TSD, task‐specific dystonia; NTSD, non‐task‐specific dystonia; NMC, non‐manifesting carriers [Colour figure can be viewed at wileyonlinelibrary.com]

This review demonstrates that structural imaging of dystonia cohorts remains a relatively under‐researched field, with much of the existing literature relying on small cohorts with methodological differences between studies, hindering reproducibility and potentially contributing to conflicting findings, together with the use of non‐biologically specific techniques (Appendix S3). Despite this variability, common themes can be seen, including changes in dMRI measures in WM pathways involved in motor control, notably between the cerebellum, brainstem, basal ganglia/thalamus and sensorimotor cortex (Figure 4). These predominantly involved lower FA, often with a corresponding higher MD. Findings from volumetric studies are more variable but have likewise implicated differences in motor regions most consistently (variably higher or lower), and longer T2* times in basal ganglia regions again point to this region being of importance. There is some indication that the nature of these abnormalities varies depending on the body part affected, underlying genotype and whether the dystonia is task‐specific, although to date the number of studies is few. Although animal models aid in providing biological understanding to the imaging signals observed, only four such studies have been undertaken in DYT1 transgenic models, implicating changes in the sensorimotor cortex, basal ganglia and cerebellum, with opposing differences observed in KO versus KI models.

Human studies of Mendelian inherited forms of dystonia have consistently implicated regions including WM deep to the sensorimotor cortex, cerebellar WM projections, brainstem and basal ganglia GM, most consistently showing lower FA (Figure 4a). Some differences appear independent of the manifestation of motor symptoms, with NMC exhibiting changes to motor WM pathways to a lesser degree than in clinically manifesting forms. Amongst sporadic dystonia cohorts results were more variable but do demonstrate widespread motor pathway abnormalities, most commonly lower FA, involving the WM between the brainstem, cerebellum, basal ganglia/thalamus and sensorimotor cortex, and higher GM volumes in the supplementary/secondary motor regions (Figure 4b). There are also indications of differences in task‐ and non‐task‐specific forms of dystonia, with a tendency towards larger volumes in implicated regions in task‐specific forms (Figure 4c). There is also evidence of reversal of some differences in relation to botulinum toxin treatment, potentially indicative of network changes being reactive or compensatory to the underlying pathophysiological mechanisms. None of these studies provides insight into the direction of cause and effect, although those involving MCs and NMCs provide opportunity for gene‐specific and motor‐independent findings [9, 10, 60].

The consistent structural findings overlap with those identified using other imaging modalities. For example, fMRI studies have identified alterations in the blood oxygen level dependent (BOLD) signal in the basal ganglia, cerebellum and sensorimotor cortex, key motor regions implicated in volumetric and relaxometry studies, and connected by WM pathways in which dMRI abnormalities have been identified [87]. Magnetoencephalography in writer's cramp has shown lower post‐movement‐event‐related synchronization of beta activity, potentially indicating impaired deactivation of the motor cortex [6], GABA spectroscopy has shown reduced GABA levels in the sensorimotor cortex and lentiform nuclei [88] and positron emission tomography with flumazenil (which binds to GABA‐A receptors) showed reduced binding in the cerebellum and sensorimotor cortex in focal hand dystonia [7], indicating that inhibitory changes may be involved in pathogenesis.

Contribution to bias from selection of participants potentially impacted a subset of studies, with relatively small sample sizes [24, 32, 34, 38, 40, 43, 49, 64, 65, 68]. Very few studies considered the non‐motor phenotype of participants which may confound results, and likewise sub‐optimal imaging methodology has the potential to introduce performance bias [9, 10, 11, 12, 13, 53, 65]. Measurement of exposure was generally adequate, with selection of participants based on diagnosis by a specialist. Blinding of outcome measures was not commented on but has less relevance with studies almost exclusively using automated ROI selection and analysis approaches. Attrition bias was relevant only to studies collecting data at multiple timepoints [20, 42, 43], with one study having substantial dropout in the 5‐year follow‐up (seven out of 19) [20]. There was no overt evidence of selective outcome reporting in the literature.

This review particularly serves to highlight the methodological limitations of many dystonia structural imaging studies to date. Notably, lower field strength and large anisotropic voxels limit a number of studies, resulting in potential compromise in data quality compared to higher resolution acquisitions. Whilst most studies did employ the key basic pre‐processing steps, other steps aimed at reducing artifacts and distortions would probably further improve data quality. In particular, few studies reported outlier detection and rejection, relevant for movement disorder cohorts in identifying and removing from analysis any acquisitions with substantial signal dropout, which could lead to false conclusions regarding systematic between‐group difference. There was also generally no documented correction for inhomogeneity in magnetic or radiofrequency fields which can particularly influence T2* relaxometry and MTR imaging respectively. The approach taken to data analysis also varied substantially, with several studies using a less hypothesis‐driven whole brain approach; additionally some of the ROI‐based studies only did so following a whole brain analysis. Whilst this approach allows for additional information to be gathered regarding the identified regions, it does reduce the validity of these as hypothesis‐driven areas of interest. An additional consistent limitation is the use of biologically non‐specific measures, such as FA, MD, volume, T2*, MTR, which do not necessarily enable inference of the nature of any underlying abnormality, aetiology or pathophysiological process (Figure 2.2).

Of the range of potential imaging approaches available, the majority of the studies focused on WM diffusion or GM volume‐based approaches, with only a small number utilizing other approaches such as relaxometry and magnetization transfer imaging. Amongst the applied approaches, there is substantial scope for application of more advanced or optimized methodologies, to enhance the level of biological meaning attributable to measured signals and provide insights into any potential role for impaired neurodevelopmental processes resulting in differences in cellular morphology. For example, newer methods for analysing diffusion data overcome several of the technical limitations, such as tractometry (involving segmentation and subsequent analysis of WM tracts), fixel‐based analysis (determines fibre‐specific measures within a single voxel) and biophysical models that attribute diffusion signals to particular underlying tissue properties. More biological specificity could be attained using techniques such as multi‐shell dMRI (collecting data at multiple diffusion gradient strengths), relaxometry utilizing multi‐exponential T2 decay and quantitative magnetization transfer methods.

## CONCLUSION

Magnetic resonance imaging differences are evident between dystonia cohorts and unaffected controls in the examination of both genetic and idiopathic forms of dystonia, although the nature of the underlying brain tissue differences remains to be established. Future dystonia imaging studies would be enriched by the recruitment of substantially larger, more deeply phenotyped cohorts—with consideration of the breadth of motor and non‐motor manifestations of dystonia and to take advantage of advanced processing and analysis techniques to enhance data quality. Further stratification of cohorts across the breadth of clinical presentation may give insight into the morphological differences underlying this heterogeneity and the associated degree of conflict in the findings in the existing literature. The gain from improved understanding of the human in vivo network‐based changes involved in instigating and driving dystonia pathophysiology provides opportunity to explore improved disease‐modifying and curative therapeutics, both directly and through more focused evaluation of other disease models. Identification of robust imaging measures may also be of benefit as non‐invasive measures of therapeutic responses in clinical trials.

## AUTHOR CONTRIBUTIONS

**Claire Maclver:** Conceptualization (equal); formal analysis (lead); funding acquisition (equal); investigation (lead); methodology (equal); visualization (equal); writing – original draft (lead); writing – review and editing (equal). **Chantal Tax:** Conceptualization (equal); funding acquisition (equal); methodology (equal); supervision (equal); writing – review and editing (supporting). **Derek Jones:** Conceptualization (supporting); funding acquisition (equal); methodology (supporting); supervision (equal); writing – review and editing (supporting). **Kathryn J Peall:** Conceptualization (equal); formal analysis (supporting); funding acquisition (equal); methodology (equal); supervision (equal); writing – review and editing (lead).

## FUNDING INFORMATION

This work was supported by an ABN/Guarantors of Brain Clinical Research Training Fellowship (520286) and a Wellcome Trust Translation of Concept Scheme (Institutional Translational Partnership Award) (520958).

## CONFLICT OF INTEREST

No conflict of interest to declare.

## FINANCIAL DISCLOSURE

No financial disclosures to declare.

## CONSENT STATEMENT

Not applicable.

## APPROVAL STATEMENT

This work was not original research but a review of the literature; therefore ethical approval was not required.

## Supporting information


Appendix S1

Appendix S2

Appendix S3

## Data Availability

N/A.
