# Supplementary figures and images for: Structural magnetic resonance imaging in dystonia: A systematic review of methodological approaches and findings
Source: Eur J Neurol. 2022 Jul 22;29(11):3418–48. doi: 10.1111/ene.15483 (PMC9796340; doi:10.1111/ene.15483)

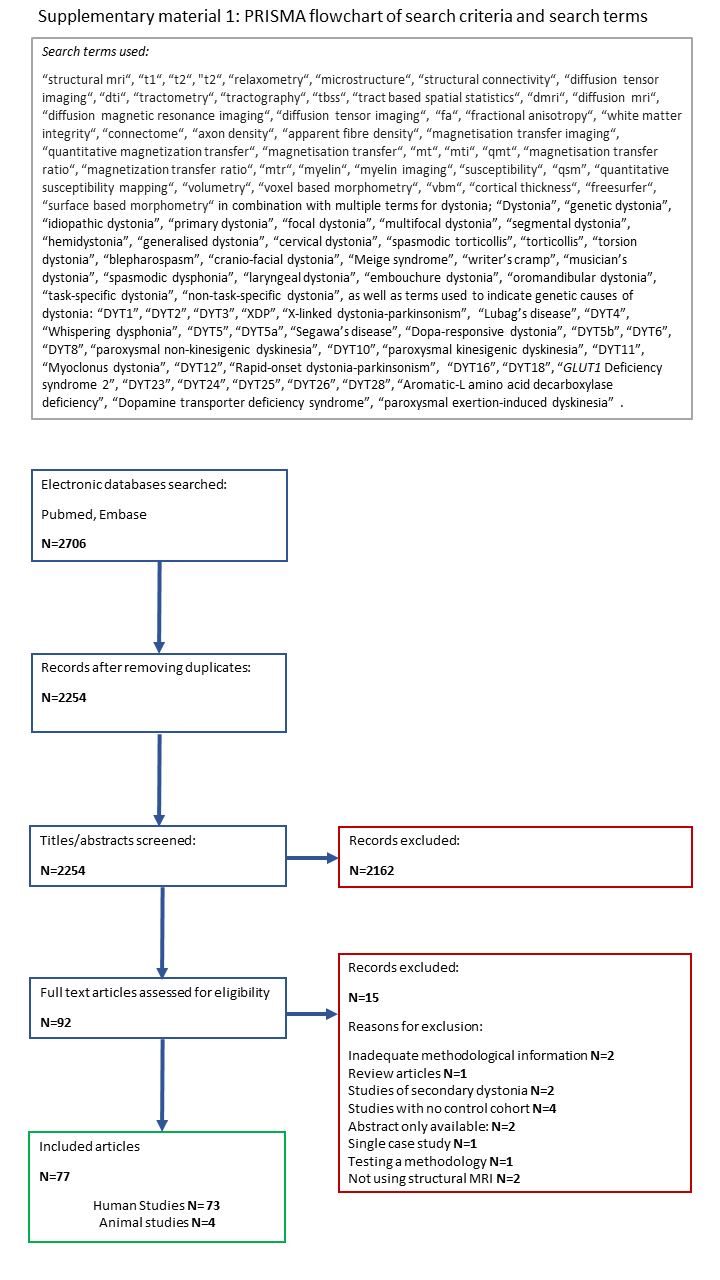

Supplement: Supplementary file 1 — Appendix S1 [file ENE-29-3418-s002.tif]
