# Supplementary material for: Structural magnetic resonance imaging in dystonia: A systematic review of methodological approaches and findings
Source: Eur J Neurol. 2022 Jul 22;29(11):3418–48. doi: 10.1111/ene.15483 (PMC9796340; doi:10.1111/ene.15483)
Supplement: Supplementary file 2 — Appendix S2 [file ENE-29-3418-s001.docx]

| **Diffusion based methodologies** | | | | | | | | | | | |
| --- | --- | --- | --- | --- | --- | --- | --- | --- | --- | --- | --- |
| **Author** | **Clinical cohort** | **Field strength** | **Diffusion directions (orientations if stated)** | **B value** | **Voxel size (mm x;y;z)** | **preprocessing** | **Diffusion model (software used/estimation method if stated)** | **Diffusion data analysis** | **ROI/Whole brain** | **ROI placement** | **Correction for multiple comparisons** |
| Carbon, 2004^9^ | DYT1 | 1.5 | 6 | 860 | 1.875;  1.875;  5 | Eddy current correction | DTI | voxel based FA analysis | Both | ROIs Identified on whole brain analysis, plus: corpus callosum, cingulate, longitudinal superior fascicle, frontooccipital fascicle, internal capsule. | Statistical threshold for FA p=0.005 and extent threshold 50 voxels. Group comparisons p=0.01. |
| Carbon, 2008^10^ | DYT1/6 | 1.5 | 6 | 860 | 1.875;  1.875;  5 | Eddy current correction, visual inspection | DTI | voxel based FA analysis | Both | ROIs identified on whole brain analysis | P<0.005 and extent threshold 10 voxels for FA |
| Delmaire, 2009^11^ | Writer’s cramp | 1.5 | 6 | 900 | 2.97;  2.97;  3 | Eddy current correction | DTI | Voxel based FA analysis; probabilistic tractography | Both | ROIs identified on whole brain analysis used as tractography seed points | P<0.01 FDR correction |
| Colosimo, 2005^12^ | Cervical | 1.5 | 6 | 1000 | 1.95;  1.95;  5 | Not commented on | DTI | ROI based FA and MD measurement | ROI based | 17 ROIs in: corpus callosum; posterior limb of internal capsule, cerebellar WM, basal ganglia, SMA. | No correction for multiple comparisons |
| Fabbrini, 2008^13^ | Cervical, blepharospasm | 1.5 | 6 | 1000 | 1.953;  1.953;  5 | Not commented on | DTI | ROI based FA and MD measurement | ROI based | 23 ROIs: corpus callosum, posterior internal capsule, basal ganglia, substantia nigra, SMA, prefrontal, premotor and primary motor cortices | No correction for multiple comparisons |
| Kostic, 2016†^14^ | Spasmodic dysphonia | 1.5 | 65 | 1000 | 2;  1;  2.6 | Rohde et al 2004 pre-processing methodology  Eddy current and motion correction | DTI (FSL DTIFIT)  Surface based morphometry | TBSS and basal ganglia mean DTI measures | Both | basal ganglia as a grey matter ROI | Permutation-based comparison. P<0.05; threshold free cluster enhancement. Between group FDR correction p<0.05. |
| Prell, 2013†^15^ | Cervical | 1.5 | not stated | not stated | not stated | Motion correction | DTI  VBM  MTI | Voxel based FA and ADC | Both | cerebellum, occipital lobe, para/hippocampal region, frontal lobe, parietal lobe, temporal lobe, precuneus | No correction for multiple comparisons |
| Bruggemann, 2016^32^ | DYT12 | 3 | 32 | 1000 | 2;  2;  2 | FSL FMRIB  Motion and eddy current correction | DTI (FSL DTIFIT) | TBSS | Whole brain | N/A | Permutation based comparison (10,000 permutations per test)  Threshold free cluster enhancement method with FWE p=0.05 |
| Bianchi, 2017†^31^ | Spasmodic dysphonia | 3 | 60 | 1000 | 1.875;  1.875;  2 | FSL FBRMIB  Motion and eddy current correction | DTI (FSL DTIFIT)  CT | TBSS | Whole brain | N/A | FWE correction p 0.01 |
| Jochim, 2018^49^ | DYT27 | 3 | 64 | 1400 | 1.81;  1.81;  2 | ExploreDTI v4.8.3. Cardiac gating,  motion and eddy current correction | DTI (FSL DITFIT) | TBSS and probabilistic tractography | Both | ROI pairs: thalami to ipsilateral precentral and postcentral gyri; thalami to dentate nucleus and pallidum to dentate nucleus. | Bonferroni correction for multiple comparisons. |
| Li, 2019†^33^ | Paroxysmal kinesigenic dyskinesia | 3 | 64 | 700 | 2;  2;  2 | FSL FMRIB v5.0  Eddy current correction | DTI (FSL DTIFIT)  VBM | TBSS and ROIs | Both | Areas identified as showing significant differences with TBSS | Permutation based comparison with threshold-free cluster enhancement. FWE correction |
| Bonilha, 2009^34^ | Cervical | 3 | 15 | 1000 | 2.5;  2.5;  2.5 | FSL FMRIB  Eddy current correction | DTI (FSL DTIFIT) | Probabilistic tractography | ROI based | Seed mask: thalamus, waypoint: middle frontal gyrus | FDR corrected p<0.05 |
| Battistella, 2018^35^ | Spasmodic dysphonia | 3 | 60 | 1000 | 2;  2;  2 | Motion and Eddy current correction | DTI (FSLFDT Bedpostx) | probabilistic tractography | ROI based | Bilateral insula, superior temporal gyrus, inferior frontal gyrus, laryngeal/orofacial primary motor cortex. | Correction for multiple comparisons (method not stated) |
| Mantel, 2020^50^ | Embouchure dystonia | 3 | 64 | 1400 | 1.81;  1.81;  2 | ExploreDTIv4.8.6. Eddy current, motion and susceptibility correction. | DTI (BEDPOSTx; DTIFIT) | probabilistic tractography | ROI based | ROI pairs: Superior parietal lobe to SMA; primary facial somatosensory and primary facial motor; SMA and primary facial motor; putamen and all cortical ROIs | Permutation based comparison (Freedman-Lane approach). FDR correction |
| Blood, 2018^36^ | DYT12 | 3 | 32 | 1000 | 2;  2;  2 | FSL FRMIB  motion and eddy current correction | DTI (FSL DTIFIT) | Probabilistic tractography; whole brain FA and MD | Both | Seeds: precentral and postcentral sulci and gyri, SMA, orbitofrontal cortex, cingulate cortex, anterior insula  Endpoints: putamen and caudate | Bonferroni correction |
| Blood, 2012^37^ | Cervical | 3 | 60 | 700 | 2;  2;  2 | FSL FMRIB v2.0  motion and eddy current correction | DTI (FSL DTIFIT) | Voxel-wise FA and MD; Probabilistic tractography | Both | Seed ROIs (manually drawn): left and right pallidum, left and right Ansa lenticularis | Bonferroni correction; Peak t values for minimum cluster of 72 voxels |
| Merchant, 2020^38^ | Writer’s cramp | 3 | 35 | 1100 | 2;  2;  2 | TORTOISE and FATCAT  Motion and eddy current correction | DTI (FATCAT- non-linear tensor estimation) | probabilistic tractography | ROI based | Tractography between each ROI pair: motor cortex, premotor ventral cortex, dorsal inferior parietal lobule, anterior inferior parietal lobule. | No multiple comparison correction |
| Berndt, 2018^51^ | Writer’s cramp | 3 | 64 | 1400 | 1.81;  1.81;  2 | ExploreDTI 4.8.3. Motion, eddy current and susceptibility correction | DTI (non-linear regression procedure) | probabilistic tractography FA and linear anisotropy calculated. | ROI based | ROIs (automated, atlas based): Seed ROI: MFG; waypoint ROIS: basal ganglia, thalamus, primary motor cortex, primary sensory cortex. | Bonferroni correction |
| Sondergaard, 2021^39^ | Cervical | 3 | 70 | 1500 | 2;  2;  2 | Distortion, eddy current and motion correction | DTI | DTI metric calculation along a tract template | ROI based | tractography based template of the dentatorubrothalamic tract | Hochberg FDR correction |
| Bonilha, 2007^40^ | Cervical | 3 | 15 | 1000 | 2.5;  2.5;  2.5 | FSL FMRIB  Eddy current correction | DTI (FSL FDT) | Voxel wise FA and MD; ROI analysis | Both | ROIs: thalamus, caudate, putamen, pallidum, precentral area, supplementary motor area; plus ROIs for the white matter between basal ganglia structures. | FDR p<0.05 correction |
| Kim, 2015†^52^ | Paroxysmal kinesigenic dyskinesia | 3 | 30 | 1000 | 1.8;  1.8;  3 | FSL FMRIB  Eddy current and motion correction. | DTI (FSL FDT)  CT  Subcortical volumetry | TBSS and ROI analysis | Both | subcortical grey matter structures | Permutation based comparison (5000 permutations), threshold-free cluster enhancement |
| Pinheiro, 2015^53^ | Cervical, blepharospasm, oromandibular | 3 | 32 | 1000 | 1;  1;  2 | FSL FMRIB v 4.1.4. Eddy current correction. | DTI (FSL FDT) | TBSS; ROI analysis; deterministic tractography | Both | Based on TBSS findings | Permutation based analysis. No multiple comparison correction |
| Horovitz 2012†^30^ | Blepharospasm | 3 | 33 | 1000 | 0.937;  0.937;  2.4 | TORTOISE Motion, eddy current and EPI distortion correction | DTI (TORTOISE software- Non-linear least squares)  GM volumes | TBSS, probabilistic tractography | Both | left face portion of precentral gyrus (identified from VBM differences), mid pons waypoint mask (to track corticobulbar tract) | No multiple comparison correction |
| Kirke, 2017†^54^ | Spasmodic dysphonia | 3 | 60 | 1000 | 0.937;  0.937;  2.4 | FSL FDT-  Motion and eddy current correction | DTI (FSL FDT-non-linear fitting)  VBM | TBSS, probabilistic tractography | Whole brain | N/A | FWE-correction p≤0.05. |
| Ramdhani, 2014†^55^ | Task-specific, non-task-specific | 3 | 33 | 1000 | 1.9375;  1.9375;  2.4 | FSL  Motion and eddy current correction | DTI (FSL FDT)  VBM | TBSS | Whole brain | N/A | No multiple comparison testing |
| Simonyan, 2008^56^ | Spasmodic dysphonia | 3 | 33 | 1000 | 0.9375;  0.9375;  2.4 | Motion and eddy current correction | DTI | TBSS and ROI based approach | Both | genu and posterior internal capsule, the mid cerebral peduncle, pyramid, lentiform nucleus, ventral thalamus, middle cerebellar peduncle, cingulum. | No multiple comparison testing |
| Guo, 2020†^57^ | Blepharospasm | 3 | 64 | 1000 | 2;  2;  3 | PANDA software Motion and eddy current correction | DTI (FSL DTIFIT)  CT | TBSS- Local diffusion homogeneity | Whole brain | N/A | Permutation based comparison |
| Tomic, 2020†^58^ | Task-specific, non- task-specific | 3 | 65 | 1000 | 2;  2;  2.6 | Rohde et al. 2004 algorithm  Motion and Eddy current correction | DTI (FSL DTIFIT)  CT  VBM | TBSS | Whole brain | N/A | permutation-based comparison. Statistical map threshold p<0.0125, threshold free cluster enhancement |
| Guo, 2021^59^ | Blepharospasm | 3 | 64 | 1000 | 2;  2;  3 | PANDA toolbox  Motion and eddy current correction | DTI- (PANDA) | deterministic tractography  Graph theory analysis | Whole brain | N/A | Bonferroni correction |
| Long, 2017^41^ | Paroxysmal kinesigenic dyskinesia | 3 | 30 | 1000 | 1.5;  1.5;  1.5 | FSL FDT  Motion and Eddy current correction | DTI (FSL FDT) | probabilistic tractography  Regional thalamic DTI | ROI based | Thalamic seeds to cortical masks | Within thalamus voxel-wise comparison FDR correction q<0.05 |
| Argyelan, 2009^60^ | DYT1/6 | 3 | 55  (Isotropically distributed) | 1000 | 0.9;  0.8;  1.8 | FSL FMRIB | DTI (FSL FDT) | probabilistic tractography | ROI based | Bilateral superior cerebellar peduncle and brainstem (pedunculopontine nucleus). Automated ROIs | Voxel level threshold p<0.001, cluster level p<0.05 |
| Fujita, 2018^61^ | DYT1, DYT6, idiopathic | 3 | 33 | 800 | 0.9;  0.9;  2.5 | FSL FMRIB | DTI (FSL FDT) | ROI; whole brain FA and deterministic tractography | Both | Deep cerebellar white matter | FA: p<0.01 uncorrected, p<0.05 cluster level corrected  Tractography: No multiple comparison correction |
| Vo, 2015a^62^ | DYT1, DYT6, idiopathic | 3 | 33 | 800 | 0.9;  0.9;  2.5 | FSL FRMRIB - Motion and eddy current correction | DTI (FSL FDT) | FA, deterministic tractography | ROI based | fMRI to identify hand/foot motor ROIs for FA and thalamic and motor cortex ROIs for tractography | Bonferroni correction |
| Li, 2020^63^ | Paroxysmal kinesigenic dyskinesia | 3 | 64 | 1000 | 0.94;  0.94;  3 | PANDA/FMRIB  Motion and eddy current correction | DTI (FSL DTIFIT)- | deterministic tractography, graph theory analysis | Whole brain | N/A | Permutation based comparison, FWE p<0.05 correction |
| Sako, 2015^64^ | DYT1 | 3 | 33 | 800 | 0.9;  0.9;  2.5 | FSL  Motion and eddy current correction | DTI (FSL DTIFIT) | Deterministic tractography | ROI based | cerebellum and clusters identified on fMRI to show significant differences used as seedpoint and waypoints | Bonferroni correction |
| Cheng, 2012^65^ | DYT6 | 3 | 15  (Electrostatic repulsion model) | 1000 | 0.78125;  0.78125;  3 | Functool 3.1.22.  Eddy current correction | DTI | ROI based FA and MD | ROI based | 24 ROIs (manually) along key motor pathways including sensorimotor cortex, corpus callosum, internal capsules, cerebellar peduncles. | No correction for multiple comparisons |
| Yang, 2014^66^ | Blepharospasm, oromandibular | 3 | 25 | 1000 | 1.875;  1.875;  3 | DTIstudio v2.4  Motion and eddy current correction | DTI (DTIstudio software v2.4) | FA, MD, RD- voxel based analysis | Whole brain | N/A | P<0.001 at voxel level, FWE correction at cluster level |
| Van der Meer, 2012†^67^ | DYT11 | 3 | 32 | 1000 | 1.79;  1.79;  3 | SPM8 (v 7.3.0)  Motion and eddy current correction | DTI (in house software)  WM volume | Voxel based FA and MD | ROI based | White matter mask for sensorimotor system | P<0.001 with 20 voxel extent threshold. FWE <0.05 correction |
| Vo, 2013^68^ | DYT1 | 3 | 55  (Isotropically distributed) | 1000 | 0.9;  0.9;  1.8 | FSL- FMRIB. Eddy current and motion correction | DTI (FSL-FDT) | Mean tissue compartment DTI measures | Whole brain | N/A | Bonferroni correction |
| Berman, 2018^70^ | Cervical, blepharospasm | 3 | some 32, others 30 | 1000 | Not stated | FSL- FMRIB  susceptibility, motion and eddy current correction | DT (FSL DTIFIT) | voxel wise FA and MD maps, ROI mean values | ROI based | automated ROIs- grey matter nuclei: bilateral: caudate, putamen, globus pallidus externus, globus pallidus internus, substantia nigra, red nucleus, and subthalamic nucleus | Voxel level comparison set at significance p<0.001; cluster based α < 0.05 corrected for multiple comparisons. Tukey-Kramer adjustment for FWE |
| Blood, 2019^42^ | Cervical | 3 | 60 | 700 | 2  2  2 | Brain extraction, eddy current and motion correction | DTI | FA,RadD,MD,AxD | ROI based | White matter medial to GPi | bonferroni |
| Blood, 2006^43^ | Cervical | 3 | 60 | 700 | 2  2  2 | Motion and eddy current correction | DTI | FA | ROI based | White matter between putamen/pallidum and thalamus | bonferroni |
| Bianchi, 2019†^44^ | Task specific idiopathic | 3 | 64 | 1000 | 2  2  2 | Not stated | DTI  Volumetry | TBSS | Whole grey/white matter | - | Clusterwise correction at 0.01, voxel wise 0.001 |
| Hanekamp, 2020^45^ | Task specific focal dystonia | 3 | 64 | 1000 | 2  2  2 | Motion, eddy currents, susceptibility distortions | DTI | Graph theoretical analysis | whole | - | - |
| Hanssen, 2018†^46^ | DYT3 | 3 | 32 | 1000 | 2  2  2 | For DTI: brain extraction, susceptibility correction, eddy current correction, motion correction | DTI  volumetry | probabilistic tractography | ROI based | seedpoint from region identified on VBM | FWE correction |

| **Volume/size based methodologies** | | | | | | | | |
| --- | --- | --- | --- | --- | --- | --- | --- | --- |
| **Author** | **Clinical cohort** | **methodology** | **Field strength** | **Voxel size (mm x;y;z)** | **preprocessing** | **ROI/Whole brain (or whole GM/WM)** | **ROI placement** | **Correction for multiple comparisons** |
| Bai, 2021^16^ | mixed | Volumetry- using Brainlab iPlan 3.0 navigation software | 1.5 | 0.7  0.7  2 | - | ROI based | Globus pallidus, caudate, putamen, thalamus | Bonferroni |
| Beukers, 2011^74^ | DYT11 | VBM | 3 | 1  1  1 | - | Whole grey and white matter | - | P<0.001 (extent threshold 20 voxels) |
| Cerasa, 2014^75^ | Dystonic tremor | VBM and cortical thickness | 3 | 1  1  1 | Bias correction,  Default vbm toolbox | whole | - | FWE for VBM (plus exploratory 0.001), for CT spatial clusters of thickness differences |
| Delnooz, 2015^76^ | cervical | VBM | 3 | 1  1  1 | Default vbm toolbox | whole | - | Cluster level correction p<0.05 |
| Egger, 2007^17^ | Mixed idiopathic | VBM | 1.5 | 0.9  0.9  1.5 | - | whole | - | FDR |
| Etgen, 2006^18^ | blepharospasm | VBM | 1.5 | 1  1  1 | - | whole | - | P<0.001 |
| Granert, 2011^80^ | Pianists focal hand dystonia | VBM | 3 | 1  1  1 | VBM5 toolbox standard processing | both | putamen | FWE |
| Hanganu, 2016^29^ | blepharospasm | Cortical thickness- using Freesurfer 5.3 image analysis suite | 3 | Not stated. Slice thickness 1mm | Motion correction, averaging of multiple volumetric T1-weighted images, removal of non-brain tissue | whole | - | P>0.001/Bonferroni/cluster-based adjustment |
| Hanssen, 2019†^69^ | DYT3 | VBM, relaxometry | 3 | 1  1  1 | Motion correction | ROI based | putamen | FWE |
| Li,2021b^77^ | PKD | GM morphological network matrices based on VBM | 3 | 1  1  1 | VBM standard processing | whole | - | Benjamin Hochberg FDR correction |
| Liu, 2020^78^ | Meige syndrome | VBM | 3 | 1  1  1 | VBM standard preprocessing | whole |  | FDR correction |
| Mantel, 2019^71^ | Embouchure dystonia | VBM and asymmetry analysis and ROI based volumetry analysis | 3 | 1  1  1 | Denoising, inhomogeneity correction, affine registration | both | Precentral and postcentral gyri and pallidum | FWE correction/ bonferroni |
| Obermann, 2007^19^ | Mixed idiopathic | VBM | 1.5 | 1  1  1 | VBM standard preprocessing | whole | - | States corrected but not methodology |
| Pantano, 2011^20^ | cervical | VBM | 1.5 | 1  1  1 | - | ROI based | primary sensory, primary motor, premotor cortices, SMA, thalamus, caudate nucleus, lentiform nucleus, and cerebellum | Cluster level and voxel level FDR correction |
| Piccinin, 2015^79^ | Mixed craniocervical | VBM | 3 | 1  1  1 | - | whole | - | FDR corrected, and additional analysis with clusters >50 voxels and p <0.001. |
| Piccinin, 2014^73^ | Mixed craniocervical | VBM and manual volumetry | 3 | 1  1  1 | - | ROI based | Cerebellum (manual and VBM) | VBM 20 voxel threshold, p<0.001 |
| Pontillo, 2020^21^ | cervical | GM volumes using SUIT version 3.4 | 1.5 | 1  1  1 | - | ROI based | Cerebellar GM volume using suit atlas, cerebellar peduncles WM, cerebellar VBM | FWE |
| Simonyan, 2012^25^ | Spasmodic dysphonia | VBM and cortical distance estimates | 3T | 0.94  0.94  1.3 | Correction for MRI inhomogeneities and noise | whole |  | Cluster extent correction/ Cluster wise correction |
| Suzuki, 2011^22^ | blepharospasm | VBM | 1.5 | 0.94  0.94  1.3 | - | whole | - | FDR |
| Vilany, 2017^72^ | Mixed craniocervical | Cortical thickness and subcortical volume (Freesurfer) | 3 | 1  1  1 | Magnetic field inhomogeneity correction | whole |  | FDR |
| Waugh, 2016^26^ | Mixed cervical and laryngeal | VBM, automated motor ROI segmentation (Freesurfer 5.1.0) and manual thalamus segmentation | 3 | 1  1.33  1.33 | - | both | Manual thalamus segmentation, automated 8 motor control regions | Bonferroni |
| Zeuner, 2015^81^ | Writers cramp | VBM | 3 | 1  0.94  0.94 | Motion correction | both | Globus pallidus and putamen | FWE |
| Draganski, 2009^23^ | mixed | VBM | 1.5 | 1  1  1 | SPM5 | whole | - | FWE |
| Draganski, 2003^24^ | cervical | VBM | 1.5 | 0.97  0.97  1.08 | SPM99  brain extraction | whole | - | P<0.001 |
| Garraux, 2004^27^ | Focal hand dystonia | VBM | 3 | 0.97  0.97  1.3 | SPM2 | whole | - | FWE |
| Martino, 2011^28^ | blepharospasm | VBM | 3 | 0.98  0.98  1.3 | SPM8 | whole | - | FWE |
| Hanssen, 2018†^46^ | DYT3 | VBM | 3 | 1  1  1 | SPM8  Motion correction | both | Basal ganglia, primary motor cortex, cerebellum | FWE |
| Prell, 2013^15^ | Cervical | VBM | 1.5 | 1  1  1 | SPM2 | whole | - | P<0.001 threshold 100 voxels |
| Li, 2019†^33^ | Paroxysmal kinesigenic dyskinesia | VBM | 3 | 1  1  1 | SPM8 | whole | - | P<0.0005 and minimum cluster size 60 voxels |
| Kim, 2015†^52^ | Paroxysmal kinesigenic dyskinesia | CT and subcortical volumetry | 3 | 1  1  1 | FreeSurfer 5.3 | whole | - | Threshold free cluster enhancement |
| Horovitz 2012†^30^ | Blepharospasm | Grey matter volume | 3 | 0.94  0.94  - | FSL-VBM software | whole | - | P<0.01, cluster size 80 voxels |
| Kirke, 2017†^54^ | Spasmodic dysphonia | VBM | 3 | 1  1  1 | VBM8 in SPM8 | whole | - | FWE |
| Ramdhani, 2014†^55^ | Task-specific, non-task-specific | VBM | 3 | 0.94  0.94  0.9 | VBM8 in SPM8 | whole | - | FWE |
| Guo, 2020†^57^ | Blepharospasm | CT | 3 | 1  1  1 | FreeSurfer 6.0.1 default parameters | whole | - | Surface based permutation testing |
| Tomic, 2020†^58^ | Task-specific, non- task-specific | CT, VBM, GM ROI volume | 3 | 1  1  1 | FreeSurfer 5.3 for CT; SPM12 for VBM, FSL FIRST for ROIs | both | Caudate, globus pallidus, putamen, thalamus, nucleus accumens, amygdala, hippocampus | FWE (and secondary VMB analysis with p<0.001 uncorrected) |
| Van der Meer, 2012†^67^ | DYT11 | WM volume | 3 | 1  1  1 | SPM8 VBM8 | ROI based | Sensorimotor system | FWE |
| Bianchi, 2019†^44^ | Task specific idiopathic | VBM | 3 | 1  1  1 | SPM12 CAT12 toolbox | whole | - | Clusterwise correction p<0.01, voxel-wise threshold p<0.001 |
| Kostic, 2016†^14^ | Spasmodic dysphonia | CT, area and volume | 1.5 | 1  1  - | Freesurfer 5.3 | whole | - | p<0.01 uncorrected |
| Bianchi, 2017†^31^ | Spasmodic dysphonia | CT | 3 | -  -  1 | Freesurfer | whole | - | Cluster level p<0.01 |

| **Other methodologies** | | | | | | | | | |
| --- | --- | --- | --- | --- | --- | --- | --- | --- | --- |
| **Author** | **Clinical cohort** | **methodology** | **Field strength** | **Voxel size (mm x;y;z)** | **preprocessing** | **Data analysis** | **ROI/Whole brain** | **ROI placement** | **Correction for multiple comparisons** |
| Gracien, 2019^47^ | Cervical | relaxometry | 3 | 1  1  1 | B1 and B0 non uniformity correction, correction for insufficient spoiling of transverse magnetisation, motion correction | T1, T2, T2*,PD maps | both | Putamen, pallidum, thalamus, caudate, mptpr cortex, cerebellum | Threshold free cluster enhancement |
| Aschermann, 2015^48^ | Cervical dystonia | R2* relaxometry | 3 | 1  1  1 | Visual inspection for artifact | R2* | ROI based | Thalamus, caudate, putamen, globus pallidus- mean values calculated | No correction for multiple comparisons |
| Hanssen, 2019†^69^ | DYT3 | relaxometry | 3 | 0.45  0.45  4 | Motion correction | T2* relaxometry | ROI based | caudate head and body, putamen, GPi, GPe, substantia nigra | FWE |
| Prell, 2013†^15^ | Cervical | MTR | 1.5 | Not stated (slice thickness 3mm) | Motion correction | MTR | both | Cerebellum, occipital lobe, para/hippocampal region, frontal lobe, parietal lobe, temporal lobe, precuneus | Whole brain analysis: P<0.001 threshold 100 voxels  ROI: FWE |

Supplementary material 2: Study imaging methodology table. FA= Fractional anisotropy; MD= mean diffusivity; RD= radial diffusivity; AD= axial diffusivity; DTI= diffusion tensor imaging; MTR= magnetisation transfer ratio; ROI= region of interest; FWE= family wise error rate; FDR= false discovery rate. † indicate studies which additionally utilise other structural methodologies detailed in the corresponding table subsection, and so with duplicate entries
