# Supplementary material for: Structural magnetic resonance imaging in dystonia: A systematic review of methodological approaches and findings
Source: Eur J Neurol. 2022 Jul 22;29(11):3418–48. doi: 10.1111/ene.15483 (PMC9796340; doi:10.1111/ene.15483)
Supplement: Supplementary file 3 — Appendix S3 [file ENE-29-3418-s003.docx]

| **Synthesis of study critique** | | | | | |
| --- | --- | --- | --- | --- | --- |
| **Author** | **Dystonia type** | **Imaging methodology used** | **Clinical characteristics summary** | **Imaging methodological summary** | **Overall risk of bias** |
| Sako, 2015 | DYT1 | Deterministic tractography | Strengths: age and gender matched  Limitations: small sample size (n=10), minimal dystonia phenotyping | Strengths: 3T, 33 diffusion directions, ROI based approach, multiple comparison correction  Limitations: voxel size 0.9mmx0.9mmx2.5mm | low |
| Vo, 2013 | DYT1 | DTI | Strengths: age and gender matched  Limitations: small sample size (n=7), no dystonia phenotyping | Strengths: 3T, 55 diffusion directions, multiple comparison correction  Limitations: voxel size 0.9mmx0.9mmx1.8mm, whole brain approach | low |
| Argyelan, 2009 | DYT1/6  MC & NMC | Probabilistic tractography | Strengths: looked into non manifesting as well as manifesting gene carriers  Limitations: no dystonia phenotyping (beyond manifesting vs non manifesting) | Strengths: 3T, 55 diffusion directions, ROI based approach, multiple comparison correction  Limitations: voxel size 0.9x0.9x1.8 | low |
| Cheng, 2012 | DYT6 | ROI based DTI | Strengths: provided some clinical characterisation  Limitations: small sample size (n=6), no age/gender data provided for controls | Strengths: 3T, ROI based approach  Limitations: 15 diffusion directions, voxel size 0.78mmx0.78mmx3mm, many ROIs compared with no multiple comparison correction, only correction for head motion and eddy currents | high |
| Carbon, 2008 | DYT1/6  MC&NMC | whole brain FA, ROI based MC vs NMC group comparison | Strengths: clinical characterisation  Limitations: small sample size (n=15 total ,7/8 in subgroup analysis) | Strengths:  Limitations: 1.5T, 6 diffusion directions, voxel size 1.88x1.88x5mm, whole brain approach identifying ROIs from this, multiple comparison correction not stringent | high |
| Carbon, 2004 | DYT1-  MC & NMC | Whole brain FA maps to identify areas for ROIs, plus prespecified ROI analysis | Strengths: some clinical characterisation  Limitations: small sample size (n=12 total, 4/8 in subgroup analysis), not gender matched | Strengths: Some ROIs prespecified  Limitations: 1.5T, 6 diffusion directions, voxel size 1.88mmx1.88mmx5mm, whole brain approach identifying ROIs from this, multiple comparison correction not stringent | high |
| Hanssen, 2018 | DYT3 | VBM with ROIs in basal ganglia and sensorimotor cortex; whole brain analysis; separate cerebellum analysis; CT and subcortical volume | Strengths: clinical characterisation  Limitations: Preliminary analysis on 10 of the patients previously published separately (Bruggemann, 2016) | Strengths: 3T, 33 diffusion directions, isotropic voxels (1x1x1mm^3^ for volumetry, 2x2x2mm^3^ for DTI), susceptibility correction in addition to motion and eddy current, some of the analysis involved predefined ROIs, multiple comparison correction  Limitations: whole brain approach in addition to ROIs | low |
| Hanssen, 2019 | DYT3 | VBM and T2* relaxometry | Strengths: clinical characterisation  Limitations: fairly small sample size (n=18) | Strengths: 3T, small voxels for volumetry  Limitations: Slice thickness 4mm for relaxometry | low |
| Van der Meer, 2012 | DYT11  (*SGCE*) | WM VBM and DTI | Strengths: some clinical characterisation  Limitations: fairly small sample size (n=16) | Strengths: 3T, restricted analysis to sensorimotor system, small voxels for VBM (1mm^3^), multiple comparison correction  Limitations: voxel size for DTI (1.79mmx1.79mmx3mm) | low |
| Beukers, 2011 | DYT11 | VBM | Strengths: clinical characterisation  Limitations: | Strengths: 3T, small isotropic voxels, analysis used age/sex/depression/anxiety/total volume (GM/WM) as covariates to account for potential confounders  Limitations: whole brain approach, multiple comparison correction not stringent | low |
| Blood, 2018 (72) | DYT12  (*ATP1A3*) | Probabilistic tractography with group differences for paralimbic vs sensorimotor regions, TBSS. | Strengths: clinical characterisation, and analysis included regression analysis for disease severity, disease duration and psychiatric symptoms  Limitations: fairly small sample size (n=17) | Strengths: 3T, 32 diffusion directions, predefined ROI based analyses, correction for multiple comparisons  Limitations: | low |
| Bruggemann, 2016 (54) | DYT12  (*ATP1A3*) | TBSS | Strengths: some clinical characterisation  Limitations: small sample size (n=10) | Strengths: 3T, 32 diffusion directions, correction for multiple comparisons  Limitations: whole brain approach | low |
| Jochim, 2018 (71) | DYT27 | whole brain TBSS, tractography between predefined ROI pairs | Strengths: commented on timing relating to botulinum toxin therapy  Limitations: small sample size (n=4) | Strengths: 3T, 64 diffusion directions, b value 1400, predefined ROIs  Limitations: | high |
| Sondergaard, 2021 | Cervical  dystonia | DTI | Strengths: large sample size(n=32), clinical characterisation (including non-motor)  Limitations: | Strengths: 3T, 70 diffusion directions, b value 1500, predefined ROIs, multiple comparison correction, susceptibility correction  Limitations: | low |
| Pontillo, 2020 | Cervical  dystonia | ROI and voxel based cerebellar volume assessments | Strengths: clinical characterisation and correlation of clinical features with MRI features  Limitations: | Strengths: Small isotropic voxels (1mm^3^), predefined ROIs, multiple comparison correction  Limitations: 1.5T | low |
| Blood, 2019 | Cervical  dystonia | FA in ROI of WM medial to globus pallidus interna measured immediately prior to botox and 4 weeks post | Strengths: clinical characterisation  Limitations: fairly small sample size (n=14) | Strengths: 3T, 60 diffusion directions, predefined ROIs, multiple comparison correction  Limitations: | low |
| Gracien, 2019 | Cervical  dystonia | T1, T2, T2* and Proton density maps; ROIs in basal ganglia, thalamus, WM, cerebellum and voxel wise whole brain | Strengths: some clinical characterisation  Limitations: fairly small sample size (n=17) | Strengths: 3T, small isotropic voxels (1mm^3^), correction for B1 and B0 non uniformity, insufficient spoiling of transverse magnetisation and motion, predefined ROIs, multiple comparison correction  Limitations: | low |
| Delnooz, 2015 | Cervical  dystonia | Whole brain VBM | Strengths: some clinical characterisation  Limitations: | Strengths: 3T, small isotropic voxels (1mm^3^),  Limitations: whole brain analysis, not very stringent multiple comparison correction | low |
| Aschermann, 2015 | Cervical  dystonia | R2* relaxometry in thalamus, caudate, globus pallidus, putamen | Strengths: some clinical characterisation  Limitations: small sample size (n=12) | Strengths: 3T, small isotropic voxels (1mm^3^), predefined ROIs  Limitations: no multiple comparison correction | low |
| Prell, 2013 | Cervical  dystonia | VBM, DTI and magnetisation transfer imaging | Strengths: clinical characterisation  Limitations: | Strengths: predefined ROIs for part of diffusion analysis, small isotropic voxels for VBM (1mm^3^)  Limitations: 1.5T, lacking methodological detail (diffusion directions, b-value, voxel dimensions for DTI and MTR), additional whole brain approach, no multiple comparisons correction for ROI analyses | unclear |
| Blood, 2012 | Cervical  dystonia | Voxel-wise FA and MD maps compared between groups. Probabilistic tractography. | Strengths:  Limitations: small sample size (n=12), 4 of the patients reported in a previous DTI study | Strengths: 3T, 60 diffusion directions, predefined ROIs, multiple comparison correction  Limitations: | low |
| Pantano, 2011 | Cervical  dystonia | VBM | Strengths: some clinical characterisation  Limitations: fairly small sample size (19, 12 had followup scan) | Strengths: small isotropic voxels (1mm^3^), predefined ROIs, multiple comparison correction  Limitations: 1.5T | low |
| Bonilha, 2009 | Cervical  dystonia | Probabilistic tractography | Strengths: some clinical characterisation  Limitations: small sample size (n=7), same participants also used in a previous DTI study, limited clinical characterisation | Strengths: 3T, predefined ROIs, multiple comparison correction  Limitations: | low |
| Bonilha, 2007 | Cervical  dystonia | i) Voxel-wise whole brain comparison of FA and MD, ii) GM and WM FA and MD, iii) ROI based analysis. | Strengths: some clinical characterisation  Limitations: small sample size (n=7) | Strengths: 3T, predefined ROIs, multiple comparison correction  Limitations: | low |
| Colosimo, 2005 | Cervical  dystonia | ROI based assessment of FA and MD | Strengths: some clinical characterisation  Limitations: fairly small sample size (n=15) | Strengths: predefined ROIs  Limitations: 1.5T, 6 diffusion directions, large anisotropic voxels (1.95mmx1.95mmx5mm). 17 ROIs with no multiple comparison correction | high |
| Draganski, 2003 | Cervical  dystonia | VBM | Strengths: clinical characterisation  Limitations: small sample size (n=10) | Strengths: Small almost isotropic voxels (0.98mmx0.98mmx1.08mm)  Limitations: 1.5T, whole brain approach, multiple comparison correction not stringent | high |
| Merchant, 2020 | Writer’s cramp | DTI | Strengths: some clinical characterisation  Limitations: small sample size (n=9), large difference in mean ages between patients and controls | Strengths: 3T, 35 diffusion directions, predefined ROIs  Limitations: no multiple comparison correction | high |
| Berndt, 2018 | Writer’s cramp | Probabilistic tractography- Mean FA and CL | Strengths: some clinical characterisation  Limitations: fairly small sample size (n=18) | Strengths: 3T, 64 diffusion directions, predefined ROIs, multiple comparison correction  Limitations: | low |
| Zeuner, 2015 | Writers cramp | VBM | Strengths: clinical characterisation  Limitations: | Strengths: 3T, small almost isotropic voxels (1mmx0.94mmx0.94mm), predefined ROIs, multiple comparison correction  Limitations: | low |
| Delmaire, 2009 | Writer’s cramp | Voxel based whole brain FA analysis and probabilistic tractography from areas of abnormality identified. | Strengths:  Limitations: minimal clinical characterisation | Strengths: multiple comparison correction  Limitations: 1.5T, 6 diffusion directions, relatively large voxels (2.98mmx2.98mmx3mm), whole brain analysis used to identify ROIs | high |
| Battistella, 2018 | Spasmodic dysphonia | Probabilistic tractography. Used the insula inputs to create subdivision to assess insula spatial organisation. | Strengths:  Limitations: small sample size (n=12), minimal clinical characterisation | Strengths: 3T, 60 diffusion directions, predefined ROIs  Limitations: multiple comparison correction approach not stated to assess rigor | low |
| Kirke, 2017 | Spasmodic dysphonia | VBM and DTI | Strengths: large sample size (n=40), some clinical phenotyping  Limitations: smaller sample sizes for subgroup analyses which were the main focus (n=20) | Strengths: 3T, 60 diffusion directions, multiple comparison correction, for VBM small isotropic voxels (1mm^3^)  Limitations: whole brain approach | low |
| Kostic, 2016 | Spasmodic dysphonia | TBSS, cortical thickness surface area and volume measures, basal ganglia DTI and volumetric measures | Strengths: some clinical characterisation  Limitations: small sample size (=13) | Strengths: 65 diffusion directions, one predefined ROI, multiple comparison correction  Limitations: 1.5T, most of analysis focussed on whole brain approach | low |
| Simonyan, 2012 | Spasmodic dysphonia | VBM and cortical thickness | Strengths: large sample size (n=40)  Limitations: minimal clinical characterisation | Strengths: 3T, correction for noise and inhomogeneities, multiple comparison correction  Limitations: whole brain approach | low |
| Simonyan, 2008 | Spasmodic dysphonia | DTI (TBSS plus ROI based) and post mortem analysis of implicated regions | Strengths: some clinical characterisation and correlation with MR parameters  Limitations: | Strengths: 3T, 33 diffusion directions, predefined ROIs  Limitations: no multiple comparison correction | low |
| Mantel, 2020 | Embouchure dystonia | Probabilistic tractography and seed-based functional connectivity analysis | Strengths: clinical characterisation and linear regression analyses with clinical parameters  Limitations: fairly small sample size (n=16) | Strengths: 3T, 64 diffusion directions, b value 1400, susceptibility correction, predefined ROIs, multiple comparison correction  Limitations: | low |
| Mantel, 2019 | Embouchure dystonia | Whole brain VBM and ROI volumes | Strengths: clinical characterisation and linear regression analyses with clinical parameters  Limitations: | Strengths: 3T, small isotropic voxels (1mm^3^), denoising and inhomogeneity correction, predefined ROIs, multiple comparison correction  Limitations: | low |
| Granert, 2011 | Musicians dystonia (pianists) | VBM in bilateral putamen | Strengths: clinical characterisation  Limitations: small sample size (n=11) | Strengths: 3T, small isotropic voxels (1mm^3^), predefined ROI, multiple comparison correction  Limitations: | low |
| Guo, 2021 | Blepharospasm | Deterministic tractography, calculation of global and regional network measures. | Strengths: large sample size (n=41), correlation of findings with clinical features  Limitations: | Strengths: 3T, 64 diffusion directions, multiple comparison correction  Limitations: fairly large anisotropic voxels (2mmx2mmx3mm), whole brain analysis | low |
| Guo, 2020 | Blepharospasm | Whole brain local diffusion homogeneity (LDH), FA and cortical thickness | Strengths: correlation of findings with clinical features  Limitations: | Strengths: 3T, 64 diffusion directions, multiple comparison correction  Limitations: fairly large anisotropic voxels (2mmx2mmx3mm), whole brain analysis | low |
| Hanganu, 2016 | Blepharospasm | Cortical thickness analysis | Strengths: clinical characterisation  Limitations: small sample size (n=13) | Strengths: 3T, multiple comparison correction  Limitations: whole brain approach | low |
| Horovitz, 2012 | Blepharospasm | VBM and dMRI (TBSS and probabilistic tractography) | Strengths: some clinical characterisation  Limitations: small sample size (n=14) | Strengths: 3T, 33 diffusion directions, predefined ROIs for part of DTI analysis  Limitations: whole brain approach only for VBM, no multiple comparison correction | low |
| Martino, 2011 | Blepharospasm | VBM | Strengths: clinical characterisation  Limitations: | Strengths: 3T, multiple comparison correction  Limitations: whole brain analysis | low |
| Suzuki, 2011 | Blepharospasm | VBM | Strengths: reasonably large sample size (n=32), clinical characterisation and correlation with outcome measures  Limitations: | Strengths: multiple comparison correction  Limitations: 1.5T, whole brain analysis | low |
| Etgen, 2006 | Blepharospasm | VBM | Strengths: clinical characterisation  Limitations: small sample size (n=16) | Strengths: small isotropic voxels (1mm^3^)  Limitations: 1.5T, whole brain approach, multiple comparison correction not very stringent | low |
| Li, 2021b | PKD | VBM based morphological network matrices with support vector machine | Strengths: large sample size (n=87), clinical characterisation  Limitations: | Strengths: 3T, small isotropic voxels (1mm^3^), multiple comparison correction  Limitations: whole brain approach | low |
| Kim, 2015 | PKD | cortical thickness analysis and TBSS | Strengths: clinical characterisation and correlation of results with disease duration  Limitations: | Strengths: 3T, 30 diffusion directions, some predefined ROI analyses for DTI, VBM voxel size 1mm^3^, multiple comparison correction  Limitations: DTI voxel size 1.8mmx1.8mmx3mm, VBM whole brain only | low |
| Liu, 2020 | Meige Syndrome | VBM | Strengths: large sample size (n=46), clinical characterisation including psychiatric symptoms  Limitations: | Strengths: 3T, small isotropic voxels (1mm^3^), multiple comparison correction  Limitations: whole brain approach | low |
| Tomic, 2020 | Mixed- TDS (Writer’s cramp, laryngeal), NTSD (Blepharospasm, cervical) | GM volumetric measures (VBM, cortical and subcortical measurements) and WM DTI TBSS | Strengths: large sample size (total n=97  (WC n=21, SD n=15, BSM n=27, Cervical n=34), clinical characterisation including accounting for pain  Limitations: | Strengths: 3T, 65 diffusion directions, VBM small isotropic voxels (1mm^3^), VBM some predefined regional analyses, correction for multiple comparisons  Limitations: DTI whole brain approach, some of VBM whole brain results reported using less stringent multiple comparison correction | low |
| Hanekamp, 2020 | Mixed TSD (WC, laryngeal) | Graph theoretical analysis | Strengths: large sample size (n=32), clinical characterisation and assessed for clinical correlations of imaging findings  Limitations: | Strengths: 3T, 64 diffusion directions  Limitations: whole brain analysis | low |
| Bianchi, 2019 | Mixed TSD (WC, musician’s focal hand dystonia, SD, singer’s laryngeal dystonia) | VBM and TBSS | Strengths: large sample size (n=47)  Limitations: subgroup analyses fairly small sample sizes , no dystonia severity assessments | Strengths: 3T, 64 diffusion directions, VBM small isotropic voxels (1mm^3^)  Limitations: whole brain approach with less stringent multiple comparison correction | low |
| Berman, 2018 | Mixed- CD and blepharospasm | Whole brain and ROI comparison of FA and MD values | Strengths: clinical characterisation with correlation analyses for severity  Limitations: subgroup analyses small sample sizes | Strengths: 3T, 30-32 diffusion directions, susceptibility correction, predefined ROIs, multiple comparison correction  Limitations: voxel size not stated | low |
| Vilany, 2017 | Mixed- blepharospasm, CD and oromandibular dystonia | Cortical thickness and subcortical volume | Strengths: large sample size (n=49), clinical characterisation  Limitations: some subgroup analysis fairly small sample size | Strengths: 3T, small isotropic voxels (1mm^3^), magnetic field inhomogeneity correction, multiple comparison correction  Limitations: whole brain analysis | low |
| Waugh, 2016 | Mixed- CD, SD | Volumetric measure: 8 automated ROIs in motor control regions; manual ROI thalami; VBM. | Strengths:  Limitations: small subgroup sizes (CD=17, SD=8) | Strengths: 3T, predefined ROIs, multiple comparison correction  Limitations: | low |
| Pinheiro, 2015 | Mixed- CD, blepharospasm, oromandibular | TBSS, tractography and ROI based comparison | Strengths: large overall sample size, clinical characterisation  Limitations: small sample size for subgroup analyses | Strengths: 3T, 32 diffusion directions  Limitations: ROIs identified on whole brain analysis, not multiple comparison corrected | high |
| Piccinin, 2015 | Mixed- CD, blepharospasm, oromandibular | VBM | Strengths: clinical characterisation and correlation with imaging findings  Limitations: | Strengths: 3T, small isotropic voxels (1mm^3^), multiple comparison correction  Limitations: whole brain analysis | low |
| Yang, 2014 | Mixed- blepharospasm and oromandibular | whole brain voxel-based analysis of DTI metrics | Strengths: clinical characterisation and correlation with imaging findings  Limitations: small subgroup analysis | Strengths: 3T, 25 diffusion directions  Limitations: voxel size 1.8mmx1.8mmx3mm, whole brain analysis with less stringent multiple comparison correction | low |
| Ramdhani, 2014 | Mixed- TSD (writer’s cramp, laryngeal) and NTSD (CD, blepharospasm) | VBM and DTI | Strengths: large overall sample size (n=45)  Limitations: minimal clinical phenotyping | Strengths: 3T, 33 diffusion directions, for VBM small isotropic voxels (0.98mmx0.98mmx0.9mm) and multiple comparison correction  Limitations: whole brain analysis, no multiple comparison correction for DTI | low |
| Piccinin, 2014 | Mixed- CD, blepharospasm, oromandibular | Manual volumetry | Strengths: large overall cohort size (n=35), clinical characterisation and correlation with imaging measures  Limitations: Gender mix not stated | Strengths: 3T, small isotropic voxels (1mm^3^), predefined ROI approach  Limitations: | low |
| Cerasa, 2014 | Mixed (dystonic tremor- neck and limb) | VBM and cortical thickness | Strengths: clinical characterisation  Limitations: small sample size (n=12) | Strengths: 3T, small isotropic voxels (1mm^3^), multiple comparison correction  Limitations: | low |
| Fabbrini, 2008 | CD and blepharospasm | DTI ROI based comparison | Strengths: clinical characterisation  Limitations: small subgroup sample sizes | Strengths: predefined ROIs  Limitations: 1.5T, 6 diffusion directions, voxel size 1.95mmx1.95mmx5mm, no multiple comparison correction (23 ROIs) | high |
| Obermann, 2007 | Mixed- CD and blepharospasm | VBM | Strengths:  Limitations: small subgroup sizes (BSM n=11, CD n=9), minimal clinical characterisation | Strengths: small isotropic voxels (1mm^3^)  Limitations: 1.5T, whole brain approach, multiple comparison approach not clearly stated | high |
| Blood, 2006 | Mixed (CD, WC) | Bilateral ROIs to measure FA in WM between putamen/pallidum and thalamus | Strengths: clinical characterisation  Limitations: small sample size (n=6) | Strengths: 3T, predefined ROIs, multiple comparison correction  Limitations: 6 diffusion directions | high |
| Garraux, 2004 | Focal hand dystonia (WC and musicians) | VBM | Strengths: large sample size (n=36), clinical characterisation  Limitations: | Strengths: 3T, multiple comparison correction  Limitations: whole brain analysis | low |
| Li, 2020 | PKD | graph theory and network-based statistic approaches | Strengths: large sample size (total n=78), clinical characterisation and correlation with imaging  Limitations: | Strengths: 3T, 64 diffusion directions, multiple comparison correction  Limitations: voxel size 0.94mmx0.94mmx3mm, whole brain approach | low |
| Li, 2019 | PKD | TBSS, VBM and ROI (based on TBSS and VBM abnormalities) | Strengths: large overall cohort size (n=45), clinical characterisation and correlation with imaging  Limitations: small subgroup sizes (n=15) | Strengths: 3T, 64 diffusion directions, VBM small isotropic voxels (1mm^3^), multiple comparison correction  Limitations: ROIs identified on whole brain analysis | low |
| Long, 2017 | PKD | DTI between thalamus and cortical regions | Strengths: clinical characterisation  Limitations: small subgroup numbers (n=12/8) | Strengths: 3T, 30 diffusion directions, small isotropic voxels (1.5mm^3^), predefined ROIs, multiple comparison correction  Limitations: | low |
| Bianchi, 2017 | Spasmodic dysphonia | Whole brain TBSS | Strengths: large total cohort size (n=89)  Limitations: some small subgroup cohort sizes, minimal clinical characterisation | Strengths: 3T, 60 diffusion directions, multiple comparison correction  Limitations: whole brain approach | low |
| Fujita, 2018 | MC and NMC DYT1 and DYT6, and idiopathic | DTI (pathways identified by fMRI abnormalities | Strengths: large total patient cohort (n=40)  Limitations: small subgroup sizes | Strengths: 3T, 33 diffusion directions, some predefined ROI analysis  Limitations: no multiple comparison correction for tractography | low |
| Draganski, 2009 | MC and NMC DYT1 and idiopathic | VBM | Strengths: large total cohort size (n=51), clinical characterisation and regression analysis with imaging findings  Limitations: small subgroup sizes | Strengths: small isotropic voxels (1mm^3^), multiple comparison correction  Limitations: 1.5T, whole brain approach | low |
| Vo, 2015 | DYT1, DYT6 and idiopathic |  | Strengths: clinical characterisation correlated with imaging findings  Limitations: Small subgroup sizes | Strengths: 3T, 33 diffusion directions, ROIs (determined by fMRI findings), multiple comparison correction  Limitations: ROIs not defined apriori | low |
| Bai, 2021 | Mixed | Basal ganglia volume | Strengths: large cohort size (n=50), results correlated with motor phenotype  Limitations: limited clinical characterisation | Strengths: predefined ROIs, multiple comparison correction  Limitations: 1.5T | low |
| Egger, 2007 | Mixed (generalised, cervical, hand) | Whole brain VBM | Strengths: large total cohort (n=31)  Limitations: small subgroup cohorts | Strengths: fairly small voxels (0.9mmx0.9mmx1.5mm), multiple comparison correction  Limitations: 1.5T, whole brain approach | low |

Supplementary material 3: Synthesis of study critique. ROI= region of interest; MC= manifesting carrier; NMC= non-manifesting carrier; WM= white matter; GM= grey matter FA= fractional anisotropy; RadD= radial diffusivity; AxD= axial diffusivity; MD= mean diffusivity; ADC= apparent diffusion coefficient; SMA= supplementary motor area; (L)= left; (R)= right; BSM= blepharospasm; OMD= oromandibular dystonia; CD= cervical dystonia; SD= spasmodic dysphonia; WC= writers cramp; PKD= paroxysmal kinesigenic dyskinesia; HC= healthy control; TSD= task specific dystonia; NTSD= non task specific dystonia; DTI= diffusion tensor imaging; fMRI= functional MRI; VBM= voxel-based morphology; TBSS = tract based spatial statistics
